# Transcriptomics Guided Engineering of Exosome‐Encapsulated Bifunctional Nanosheets Targeting the Immune‐PI3K/Akt Axis for Osteoporosis Therapy

**DOI:** 10.1002/advs.202511327

**Published:** 2025-09-29

**Authors:** Rongze Tang, Guanhong Liu, Congcong Wang, Minghao Chao, Guangyu Ma, Pengjin Wei, Siling Du, Junjie Li, Yufan Liu, Chaonan Jing, Guoquan Wu, Ming Guan, Hongliang Chen, Fenglei Gao

**Affiliations:** ^1^ Department of Orthopedics The Affiliated Hospital of Xuzhou Medical University Jiangsu 221004 China; ^2^ Key Laboratory of New Drug Research and Clinical Pharmacy Xuzhou Medical University Jiangsu 221004 China; ^3^ Department of Laboratory Medicine, Huashan Hospital, Shanghai Medical College Fudan University Shanghai 200040 China; ^4^ Department of Orthopedics, The Affiliated Huai'an Hospital of Xuzhou Medical University The Second People's Hospital of Huai'an Huai'an 223002 China; ^5^ Department of Orthopaedic Surgery Lishui Central Hospital and Fifth Affiliated Hospital of Wenzhou Medical University Lishui 323000 China

**Keywords:** bone regeneration, inflammation, nanosheet, osteoporosis

## Abstract

Osteoporosis, characterized by imbalanced bone metabolism and chronic inflammation, remains a therapeutic challenge due to the limitations of current single‐target therapies. This study integrates transcriptomic insights with nanomaterial engineering to develop a multi‐target strategy. Transcriptomic analysis of ovariectomized (OVX) mice reveals immune dysregulation and PI3K/Akt pathway activation, driving osteoclastogenesis. To address this, cobalt‐aluminum layered double hydroxide nanosheets (f‑CA(OH)) are synthesized, which scavenge reactive oxygen species (ROS) via peroxidase‐like activity and stabilize hypoxia‐inducible factor 1α (HIF‑1α) to upregulate BMP2 expression. Co‐culturing f‑CA(OH) with mesenchymal stem cells (MSCs) generate engineered exosomes (fCA‑BExo), encapsulating BMP2 and nanomaterials. In vitro, fCA‑BExo suppress osteoclast differentiation by blocking PI3K/Akt signaling and enhance osteogenesis via SMAD2/RUNX2 activation. In vivo, fCA‑BExo restored trabecular architecture in OVX mice, reduces pro‐inflammatory cytokines, and promote M2 macrophage polarization, demonstrating biocompatibility and efficacy. This “immune‐PI3K/Akt axis” targeting strategy offers a novel paradigm for osteoporosis treatment.

## Graphical abstract

A multifunctional nanosheet–exosome hybrid system incorporating f‐CA(OH) nanosheets enriched with abundant surface vacancies are developed. These nanosheets fuse with natural exosomes, endowing them with nanozyme‐like catalytic capabilities while retaining their inherent biological functions. The resulting hybrid system integrates immunomodulatory activity, inhibition of osteoclastogenesis, and promotion of bone regeneration, offering a promising therapeutic strategy for combating osteoporosis and enhancing skeletal repair.

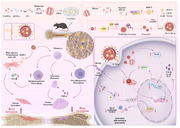

## 1 Introduction

Osteoporosis constitutes a systemic disorder of the skeleton, characterized by an^[^ ^1^ ^]^imbalance in the processes of bone metabolism,^[^ ^2^ ^]^ where declining osteogenic activity and increased osteoclastic activity^[^ ^3^ ^]^ combine to disrupt normal bone function.^[^ ^4^ ^]^ Although current clinical treatments (such as bisphosphonates and hormone therapy) can temporarily inhibit bone resorption,^[^ ^5^, ^6^ ^]^ their long‐term use is often marred by serious concomitant effects and fails to correct defects in bone formation.^[^ ^7^ ^]^ Recent research reveals that osteoporosis is associated with a bone marrow microenvironment marked by persistent low‐grade inflammation, where inflammatory mediators activate immune cells, thereby enhancing osteoclast differentiation.^[^ ^8^ ^]^ Critically, traditional anti‐resorptive and anabolic agents predominantly target single pathways (e.g., osteoclast inhibition or osteoblast stimulation),^[^ ^1^, ^9^, ^10^ ^]^ failing to address the complex interplay within the immune‐metabolism‐bone network essential for effective bone mass restoration.^[^ ^11^ ^]^ This fundamental limitation underscores the challenge of achieving sustained bone mass reconstruction with conventional approaches.

Recent advancements in high‐throughput sequencing have provided insights into the molecular mechanisms that contribute to osteoporosis. Single‐cell sequencing has revealed a profound link between immune dysregulation and bone metabolism imbalance,^[^ ^12^ ^]^ while whole‐transcriptome analyses have identified abnormal expression of the Malat1 gene in osteoclasts, leading to their excessive activation.^[^ ^13^ ^]^ Dynamic sequencing across different differentiation stages shows that the circular RNA circFam190a remains highly expressed during osteoclast development.^[^ ^14^ ^]^ These discoveries have largely expanded the pool of potential molecular targets for therapy. Additional studies have identified abnormal YAP1^[^ ^15^, ^16^ ^]^ activation in arthritis patients‐information that has spurred the design of targeted nanoparticles to inhibit the associated pathways.^[^ ^17^ ^]^ Moreover, transcriptome sequencing in osteoporosis research has highlighted that mitochondrial metabolic disorders in macrophages can worsen disease progression, prompting exploration of treatments based on engineered mitochondria delivery.^[^ ^18^ ^]^ Although these omics‐driven approaches validate the concept of nanomedicine design, significant hurdles persist: the coordinated network of immune and metabolic pathways in osteoporosis has not been fully decoded,^[^ ^19^ ^]^ and traditional single‐target drugs lack the synergistic modulation needed to disrupt the vicious cycle between inflammation and metabolism.^[^ ^20^ ^]^ These treatments also often suffer from poor bone marrow targeting and limited bioactive molecule delivery.^[^ ^21^ ^]^ In contrast, mesenchymal stem cell‐derived exosomes exhibit strong therapeutic potential^[^ ^22^, ^23^ ^]^ due to their inherent bone‐targeting ability^[^ ^24^ ^]^ and their capacity to deliver active molecules like miRNA^[^ ^25^, ^26^ ^]^ and BMP2,^[^ ^27^, ^28^ ^]^ achieving both immunomodulation and osteogenic regeneration.^[^ ^29^ ^]^ However, their clinical application is limited by low yield^[^ ^30^ ^]^ and rapid degradation in the presence of matrix metalloproteinases (MMPs) within pathological microenvironments.^[^ ^31^ ^]^ To tackle these challenges, a promising strategy involves the integration of nanomaterials with exosomes sourced from mesenchymal stem cells.^[^ ^32^ ^]^ Nanosheets, a novel 2D material, offer several biomedical advantages due to their atomic‐scale thickness and laterally extended structure, which results in an ultra‐high specific surface area.^[^ ^33^ ^]^ Their abundant surface‐active sites^[^ ^24^ ^]^ and unique electronic vacancy distributions endow f‐CA(OH) nanosheets with robust catalase‐mimicking activity,^[^ ^34^ ^]^ enabling efficient decomposition of reactive oxygen species (ROS), a key feature for mitigating oxidative stress.^[^ ^35^, ^36^ ^]^ Furthermore, the versatile surface chemistry of these nanosheets facilitates functionalization and conjugation,^[^ ^37^ ^]^ offering a platform for biomolecule loading and delivery. The double hydroxide nanomaterials (f‑CA(OH)) synthesized in this study not only retain the advantages of conventional nanosheets but also significantly enhance exosome secretion efficiency and precisely regulate the proportion of key bioactive components within exosomes.

**Scheme 1:**
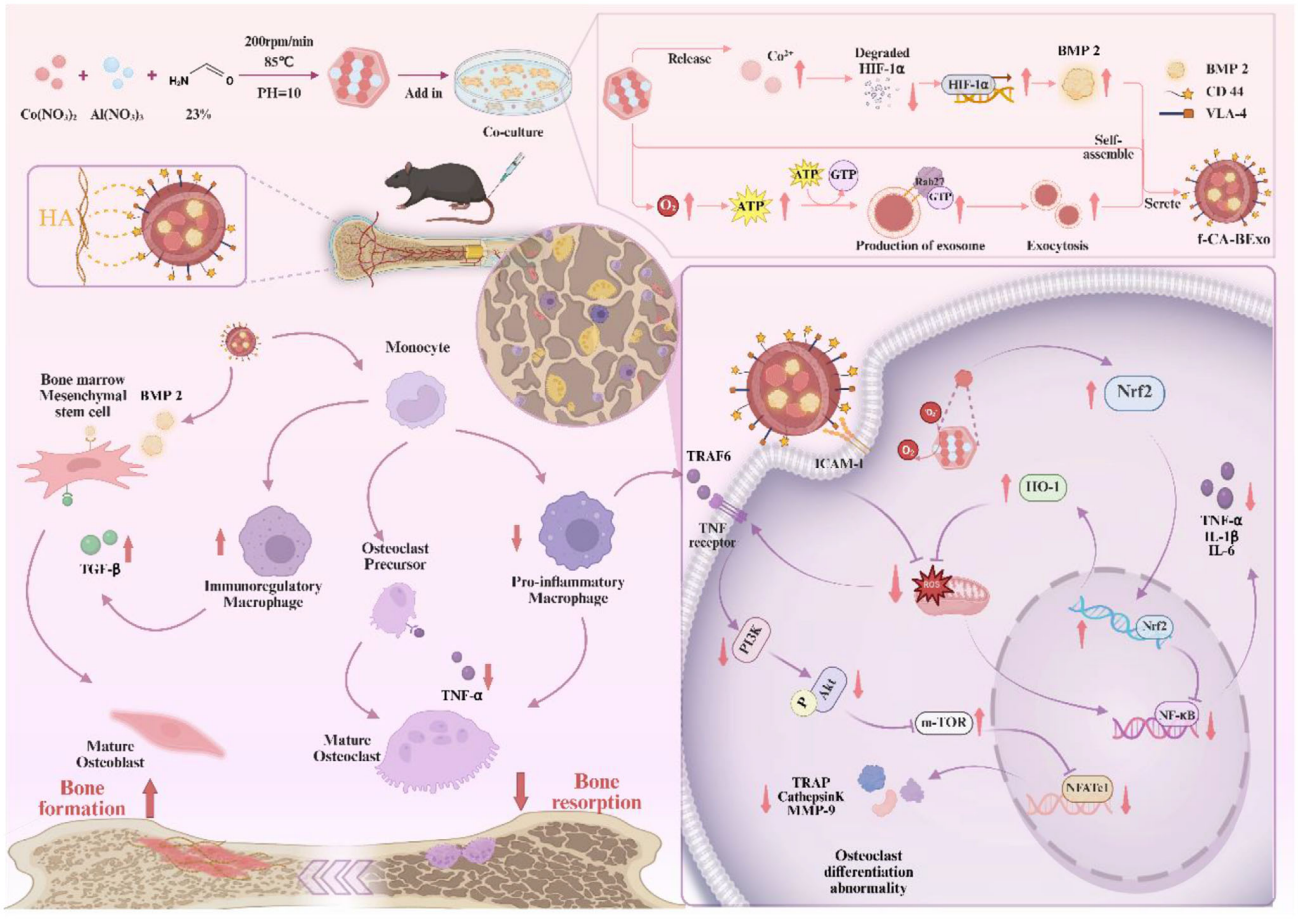
Therapeutic Process: fCA‐BExo accumulates at osteoporosis lesions via CD44‐mediated homing effects. The nanosheet component efficiently scavenges excess reactive oxygen species (ROS) through catalase‐like activity, activating the Nrf2 antioxidant pathway to mitigate mitochondrial damage. Concurrently, it suppresses hyperactivation of the PI3K‐Akt‐mTOR signaling axis, blocking NFATc1‐driven osteoclast differentiation. The exosomes release BMP2 to stimulate osteogenic differentiation of BMSCs, establishing a dynamic balance between “osteoclast inhibition and osteogenesis promotion”. (created in BioRender. 2, 1. (2025) https://BioRender.com/n78z61z).

In response to these issues, we first employed transcriptome sequencing to analyze changes in immune regulation–related signaling pathways, revealing a coordinated activation network between immune regulation and the PI3K pathway in the osteoporosis model, with ROS accumulation identified as the primary driver of the pathological cascade.^[^ ^38^, ^39^ ^]^ Building on these insights, we have developed an innovative composite system‐fCA‐BExo‐comprising functionalized cobalt‐aluminum layered double hydroxide nanomaterials (f‑CA(OH)) combined with exosomes (**Scheme** **1**). This system offers two key innovations: 1) Multi‐dimensional Synergistic Regulation Mechanism: Once targeted and delivered to the bone marrow microenvironment, The system exhibits catalase‐like activity, effectively scavenging reactive oxygen species (ROS) and thereby activating the Nrf2 antioxidant pathway to alleviate mitochondrial damage. Meanwhile, by reducing ROS accumulation, it prevents ROS‐induced hyperactivation of the PI3K‐Akt‐mTOR signaling axis, contributing to anti‐inflammatory effects and cellular homeostasis, blocking NFATc1‐mediated osteoclast differentiation to remodel the inflammatory microenvironment while inhibiting osteoclast activity. 2) Exosome Engineering Enhancement Strategy: By stabilizing HIF‐1α protein, the expression level of BMP2 in BMSC‐derived exosomes is elevated, and the activation of the RAB27a‐dependent secretion pathway markedly increases the production of engineered exosomes. In addition, a self‐assembly technique encapsulates BMP2 within the exosome–nanocomposite, and CD44‐mediated homing further reduces the risk of ectopic calcification. This homing utilizes natural CD44 on MSC‐exosomes^[^ ^40^, ^41^ ^]^ binding bone marrow hyaluronic acid (HA),^[^ ^42^, ^43^ ^]^ directing exosomes to bone surfaces and minimizing off‐target BMP2 delivery, thereby reducing ectopic calcification risk.^[^ ^44^ ^]^

The innovation of this strategy lies in its “trinity” design: using bioinformatics to pinpoint key pathological targets, guiding the synthesis of functionalized nanomaterials, and integrating their catalytic properties with BMP2's osteogenic induction through self‐assembly, all of which harness the natural carrier functions of stem cell–derived exosomes to achieve precise delivery.

## 2 Results and Discussion

### 2.1 Transcriptome Sequencing Reveals Potential Mechanisms of Immune Alterations in Osteoporotic Model Mice

**Figure 1:**
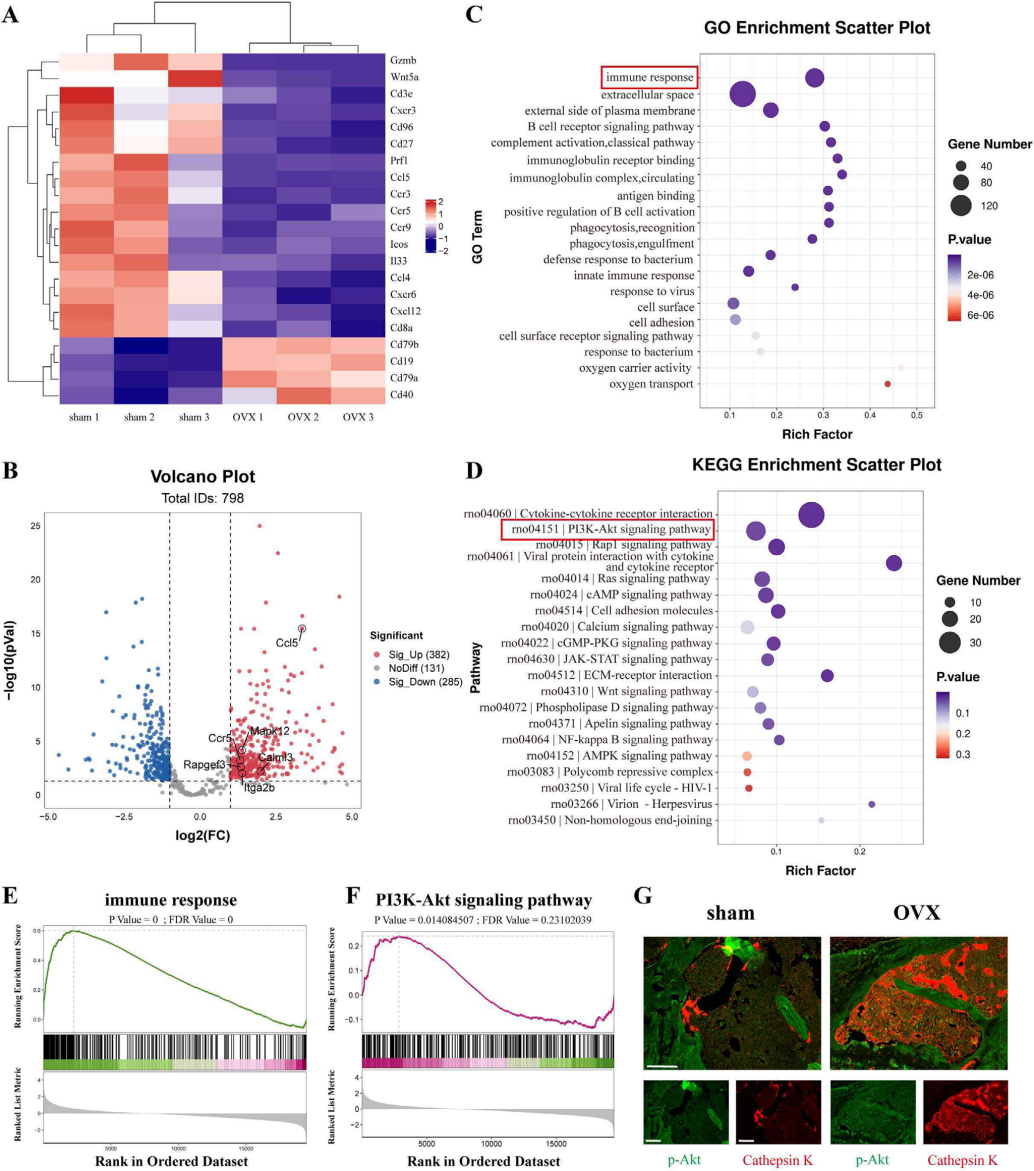
Immune Reprogramming, PI3K‐Akt Activation, and Osteoclast Expansion in OVX‐Induced Osteoporosis. A) A heatmap displaying hierarchical clustering of differentially expressed genes in femoral bone marrow between the sham and OVX groups, highlighting 382 genes that were upregulated and 285 genes that were downregulated (FDR < 0.05). B) A volcano plot illustrating differentially expressed genes, with red indicating significant upregulation, blue signifying significant downregulation, and gray representing non‐significant results. C) The Gene Ontology (GO) enrichment analysis emphasizing substantial enrichment of biological processes related to immune response in the OVX group (P = 0, FDR = 0). D) KEGG pathway analysis indicating significant enrichment in the PI3K‐Akt signaling pathway (P = 0.014, FDR = 0.231). E) Variations in GO terms related to immune responses, with an enrichment factor exceeding 5. F) A comparison of activation levels for the PI3K‐Akt pathway from the KEGG analysis, showing a 2.1‐fold increase in the OVX group. G) Immunofluorescence staining revealing the expression of Cathepsin K⁺ osteoclasts and phosphorylated AKT (p‐AKT) in femurs (scale bar: 100 µm).

Our analysis of transcriptome sequencing from the femoral bone marrow of ovariectomized (OVX) mice and sham‐operated controls uncovered significant reprogramming of immune‐related transcription associated with the osteoporotic phenotype. Hierarchical clustering analysis (**Figure** **1**A) revealed a clear distinction between the two groups, with key immune genes (Cd3e, Cd8a, and Cd79a) showing pronounced expression differences, suggesting systemic immune remodeling in OVX mice. Through volcano plot analysis (Figure 1B), we identified 382 significantly upregulated genes (for instance, Ccl5, Ccr5, and Rapgef3) and 285 downregulated genes (FDR < 0.05) in the OVX mice. Notably, the increased levels of chemokines Ccl5 and Ccr5, known to play a role in recruiting inflammatory cells, indicate a rise in immune cell infiltration linked to bone resorption.

Gene Ontology (GO) enrichment analysis (Figure 1C, E) demonstrated a highly significant enrichment of biological processes related to immune responses in the OVX group (P = 0, FDR = 0), underscoring a notable dysregulation of the immune system in cases of osteoporosis. Investigations into the underlying mechanisms indicated a substantial upregulation of cytotoxic effectors, such as perforin Prf1 and granzyme B Gzmb, along with the chemokine Ccl5 (P < 0.01). These molecular characteristics suggest an abnormal activation of both the innate and adaptive immune systems. Additionally, integrated multi‐omics data imply a cascade of dysregulation within the immune regulatory network present in the osteoporotic microenvironment. Notably, this altered immune microenvironment could interfere with the dynamic equilibrium between bone formation and resorption by facilitating osteoclast differentiation and augmenting the activity of bone resorption, ultimately leading to a pathological environment characterized by osteolysis.

The Kyoto Encyclopedia of Genes and Genomes (KEGG) pathway analysis (Figure 1D, F) revealed that the signaling pathway involving phosphatidylinositol 3‐kinase (PI3K) and protein kinase B (Akt) functions as a key hub. This analysis also indicated a simultaneous enrichment of cytokine‐cytokine receptor interactions along with Ras/cyclic adenosine monophosphate (cAMP) pathways. In mice that serve as models for osteoporosis, a moderate enrichment of the PI3K‐Akt pathway was observed (P = 0.014, FDR = 0.231) (Figure 1F), aligning with its established role in the activation and survival of osteoclasts. The synergistic dysregulation observed in this pathway, coupled with immune pathways such as cytokine‐cytokine receptor interactions, hints at its possible involvement in immune‐mediated bone loss.

Laser confocal microscopy (Figure 1G) revealed a significant increase in Cathepsin K‐positive (Cathepsin K⁺) osteoclasts (2.8‐fold, P = 0.003) in the femoral metaphysis of OVX mice, accompanied by elevated Akt phosphorylation levels (approximately 2.1‐fold). The relationship between molecular and phenotypic characteristics highlights the promise of focusing on immune interaction networks as a new therapeutic approach to counteract osteoporotic conditions.

### 2.2 Synthesis, Characterization, and Biosafety Evaluation of f‐CA(OH) Nanoparticles

To tackle the previously mentioned imbalance in the immune microenvironment and the hyperactive of the PI3K/AKT pathway, this study developed enzyme‐mimetic f‐CA(OH) nanosheets by harnessing the antioxidant properties of innovative nanomaterials. The objective was to improve the immune balance affected by oxidative stress through the effective regulation of reactive oxygen species (ROS) levels in the inflammatory microenvironment, while simultaneously decreasing the overactivation of the PI3K/AKT signaling pathway.

**Figure 2:**
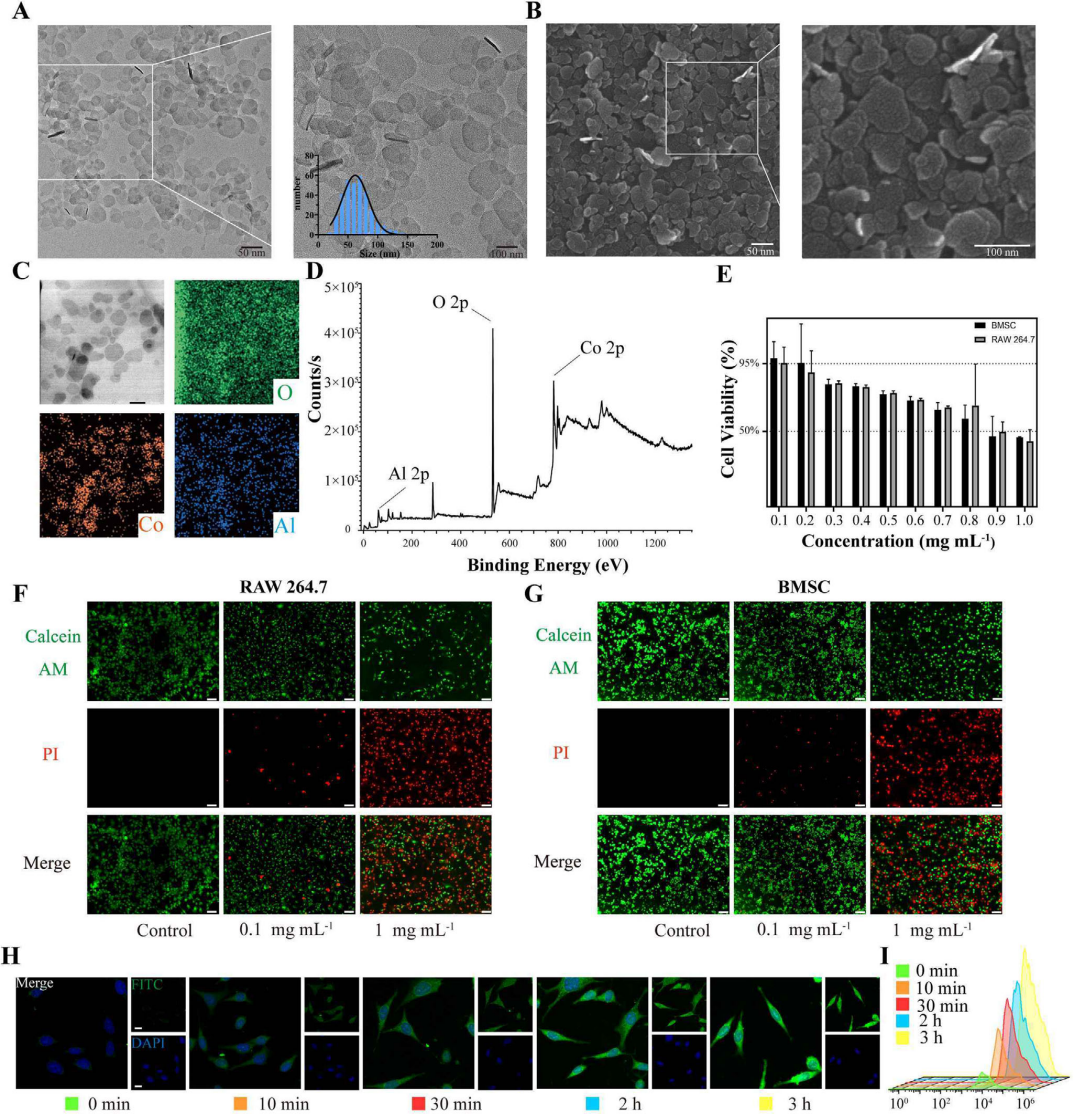
Synthesis, biosafety, and cellular uptake of f‐CA(OH) nanosheets. A) Transmission electron microscopy (TEM) image of f‐CA(OH) nanosheets and their diameter distribution. B) The scanning electron microscopy (SEM) image depicts f‐CA(OH) nanosheets. C) Elemental mapping illustrating O, Co, and Al within f‐CA(OH) nanosheets (scale bar: 50 nm). D) The spectrum obtained from X‐ray photoelectron spectroscopy (XPS) for f‐CA(OH) nanosheets E) Assessment of the viability of BMSC and RAW 264.7 cells following incubation with varying concentrations of f‐CA(OH) via CCK‐8 assay. F) Fluorescence images showing BMSC cells that were double‐stained with Calcein‐AM and PI are indicated (scale bar: 100 µm). G) Fluorescence images of RAW 264.7 cells also double‐stained with Calcein‐AM and PI (scale bar: 100 µm). H) Confocal laser scanning microscopy (CLSM) images revealing BMSC cellular uptake of FITC‐f‐CA(OH) at multiple time points (scale bar: 10 µm). I) Flow cytometry was utilized to investigate the levels of FITC‐f‐CA(OH) inside BMSC cells at various time intervals. All results are shown as mean ± SD.

Following the synthesis protocol outlined in (Scheme 1), f‐CA(OH) nanosheets were successfully prepared and characterized at multiple scales, including morphology observation, elemental composition, and chemical state analysis. In terms of morphology, TEM (**Figure** **2**A) and SEM (Figure 2B) analysis revealed that the material exhibited a typical 2D nanosheet structure, with statistical analysis of three randomly selected TEM images yielding an average diameter of 66.74 nm (Figure 2A). Elemental mapping analysis by EDS (Figure 2C) confirmed the uniform distribution and spatial co‐localization of Co, Al, and O within the nanosheets. Chemical state analysis via XPS (Figure 2D; Figures S1, S2, Supporting Information) revealed characteristic Co 2p double peaks at 797.65 and 781.55 eV and splitting peaks of Al 2p orbitals at 75.05 and 74.25 eV, consistent with the crystalline features of layered double hydroxide (LDH).

To assess the stability of the nanosheets, we monitored the physicochemical properties of f‐CA(OH) under different storage conditions (PBS and 10% FBS) over time. Notably, both conditions led to significant aggregation and sedimentation (Figures S3, S4, Supporting Information). Dynamic light scattering (DLS) analysis showed that the hydrodynamic diameter remained stable during the first 3 days but increased significantly thereafter, indicating progressive nanosheet aggregation.

Subsequently, to identify the optimal co‐cultivation concentration of f‐CA(OH), we conducted systematic cytotoxicity evaluations. The results from CCK‐8 assays (Figure 2E) revealed that the viabilities of BMSC and RAW264.7 cells markedly declined as the concentration of f‐CA(OH) escalated (ranging from 0.1 to 1.0 mg mL^−1^), particularly at higher doses (≥0.9 mg mL^−1^), where the survival rates significantly fell to 46.82% and 49.77%, respectively. This suggests a possible cytotoxic effect at increased concentrations. Conversely, at lower concentrations (< 0.1 mg mL^−1^), the effect of f‐CA(OH) on cell viability was negligible, with survival rates recorded at 98.58% and 95.35%, indicating that the toxicity remains manageable.

Membrane integrity was further validated using live/dead staining assays (Figure 2F, G; Figure S5, Supporting Information), which confirmed that under low concentrations (0.1 mg mL^−1^), Calcein‐AM (green, live cells) signals predominated, with minimal PI (red, dead cells) staining, showing no significant difference compared to the normal control group. In contrast, the PI‐positive cells increased to 49.425% (BMSC) and 50.250% (RAW264.7) at the 0.9 mg mL^−1^ concentration, significantly higher than the control group (p < 0.001). Additionally, flow cytometry analysis (Figures S6, S7, Supporting Information) indicated concentration‐dependent increases in f‐CA(OH)‐induced apoptosis. Notably, at concentrations ≥0.7 mg mL^−1^, the proportions of early and late apoptotic cells (Q3‐4 and Q3‐2 quadrants) significantly increased, further confirming that higher concentrations of f‐CA(OH) could induce apoptosis. Based on these findings, 0.1 mg mL^−1^ was established as the safe threshold for subsequent studies.

To evaluate the cellular uptake of f‐CA(OH), we employed FITC‐labeling tracing techniques in conjunction with quantitative analysis. Confocal images (Figure 2H, I) revealed time‐dependent accumulation of FITC‐labeled f‐CA(OH) (green) in cells. At 0 min, almost no fluorescence was detected, while at 30 min, flow cytometry‐based quantitative analysis showed that the fluorescence intensity of f‐CA(OH) increased approximately threefold at 30 min compared to the initial time point, peaking at 1 hour before plateauing. These results indicated that the uptake of f‐CA(OH) was time‐dependent, with a rapid early rate and major internalization occurring within 30 min. This finding supports the high cellular compatibility and efficient delivery potential of f‐CA(OH), as well as optimizing the co‐cultivation time selection.

### 2.3 Induction, Isolation, and Characterization of Customized Mesenchymal Stem Cell‐Derived Exosomes

Previous RNA sequencing data revealed that the alteration of the immune microenvironment, along with the enhancement of the PI3K/Akt signaling pathway associated with osteoclast activity, represent critical mechanisms responsible for bone loss in osteoporosis. Existing evidence demonstrates that exosomes originating from stem cells have the ability to influence macrophage polarization through the transfer of particular miRNAs, while suppressing the activation of the PI3K/Akt pathway in inflammatory cells. Based on these findings, we investigated the co‐cultivation of nanostructures with stem cells, to develop a dual‐targeting therapeutic strategy that simultaneously modifies the immune microenvironment and curtails osteoclast activity, thus facilitating a synergistic treatment outcome.

The preparation of customized mesenchymal stem cell‐derived exosomes was performed as follows. When BMSCs reached approximately 60% confluence, 0.1 mg mL^−1^ f‐CA(OH) was added to the culture medium. Once the cells achieved 90% confluence, the complete culture medium was substituted with serum‐free medium containing an equivalent concentration of f‐CA(OH), and the cells were further cultured for 12 h. Exosomes were subsequently isolated from the supernatant using gradient centrifugation.

The harvested exosomes were resuspended in 1 mL PBS, and their concentration was quantified by nanoparticle tracking analysis (NTA), revealing 1.1×10⁸ particles/mL UV–vis spectroscopy was employed to measure the absorption peak intensity of f‐CA(OH), indicating a residual concentration of 68 µg mL^−1^ in the exosomal suspension (Figure S8, Supporting Information). Based on this, the f‐CA(OH) content per exosome was calculated to be 6.18×10^−^⁷ µg.

Western blot analysis detected strong expression of exosomal markers (CD9, CD90, ALIX, and TSG101), while the mitochondrial contamination marker TOM70 was undetectable (Figure S9, Supporting Information). Transmission electron microscopy confirmed that the engineered exosomes exhibited the characteristic cup‐shaped bilayer morphology (Figure S10, Supporting Information) with an average diameter of 161.3 ± 12.7 nm (compared to 30–160 nm for conventional exosomes). Nanoparticle tracking analysis (NTA) measured the hydrodynamic diameter of 162.4 ± 15.3 nm (Figure S11, Supporting Information). The surface charge analysis revealed that the engineered exosomes had a zeta potential of −17.1 ± 1.3 mV, significantly less negative than that of conventional exosomes (−23.9 ± 0.9 mV) (Figure S12, Supporting Information), confirming the successful loading of f‐CA(OH). Further, energy dispersive spectroscopy (EDS) was employed to observe the spatial distribution of elements within the bilayer membrane structure. The exosomal membrane, enriched in C, O, N, and P, also contained the distinctive Co and Al signatures from the nanosheets, directly confirming the successful f‐CA(OH) loading (Figure S13, Supporting Information).

### 2.4 Bimetallic Nanozymes Synergize with Exosomes to Reprogram Macrophage Polarization for Osteoporosis Therapy

Under pathological conditions such as osteoporosis, oxidative stress triggers chronic activation of macrophages, promoting sustained osteoclast differentiation and excessive phosphatase production, ultimately exacerbating bone loss. Vacancy‐rich bimetallic nanosheets exhibit intrinsic nanozyme activity through catalytic decomposition of H_2_O_2_ into O_2_ and H_2_O.^[^ ^45^, ^46^, ^47^ ^]^ To evaluate f‐CA(OH)’s therapeutic potential and its synergistic effects with engineered exosomes in regulating redox homeostasis and reprogramming macrophage polarization, we conducted a systematic evaluation using multifaceted experimental approaches.

**Figure 3:**
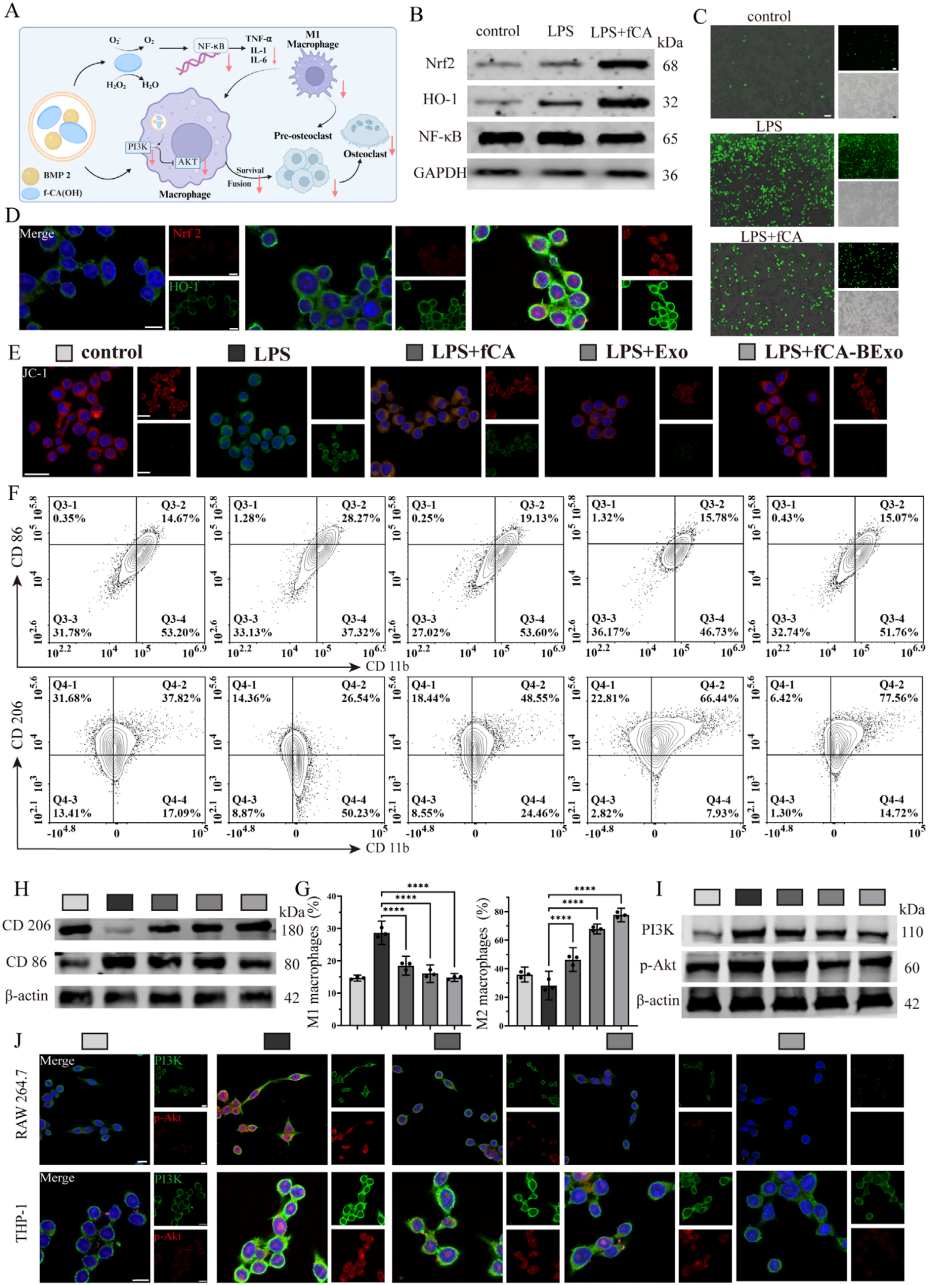
Nanozyme‐exosome synergy reprograms macrophages and blocks PI3K‐AKT in osteoporosis. A) Schematic representation illustrating the synergistic effect of fCA‐BExo on macrophage polarization and osteoclast differentiation (Created with Biorender). B) Analysis via Western blot of protein expression levels of Nrf2, HO‐1, and NF‐κB in RAW 264.7 cells following treatment with f‐CA(OH). C) Confocal microscopy images depicting ROS fluorescence in RAW 264.7 cells after f‐CA(OH) administration (scale bar: 200 µm). D) Subcellular localization and immunofluorescence expression of Nrf2 and HO‐1 in RAW 264.7 cells by CLSM (scale bar: 10 µm). E) JC‐1 fluorescence images in RAW 264.7 cells under varying experimental conditions (scale bar: 10 µm). F) Flow cytometry assessment of RAW 264.7 cell polarization status across varying conditions. G) Quantitative flow cytometry of macrophage subsets: CD11b⁺CD86⁺ (M1) and CD11b⁺CD206⁺ (M2) populations (n = 3). H) Western blot examination of protein expression for CD206 and CD86 in RAW 264.7 cells subjected to different conditions. I) Western blot evaluation of protein expression levels of PI3K and p‐AKT in osteoclast precursors subjected to varying conditions. J) Confocal laser scanning microscopy (CLSM) investigation of PI3K and p‐AKT localization at the subcellular level, including immunofluorescence expression profiles in osteoclast precursors (scale bar: 10 µm). All results are shown as mean ± SD. ns ≥0.05, **p < 0.01, ****p < 0.0001.

Initially, ROS fluorescence probe assays demonstrated that LPS stimulation, which mimics an inflammatory response, triggered an intracellular ROS burst in macrophages (Figure **3C**), while treatment with f‐CA(OH) significantly scavenged 94.6% of the excess ROS. Further Western blot analyses assessing the Nrf2/HO‐1 antioxidant signaling pathway and the activation of NF‐κB, a crucial nuclear transcription factor driving pro‐inflammatory macrophage transformation (Figure 3B), demonstrated that f‐CA(OH) strongly upregulated the Nrf2/HO‐1 pathway (with nuclear Nrf2 and HO‐1 protein expression increased by 6.5‐fold and 8.1‐fold relative to control, respectively; Figures S14, S15, Supporting Information), while concurrently inhibiting NF‐κB nuclear translocation (69.5% reduction versus LPS‐treated cells; Figure 3B; Figure S16, Supporting Information). This dual modulation simultaneously attenuated oxidative stress and inflammatory cascades. Immunofluorescence staining further confirmed Nrf2 activation in both nuclei and cytoplasm, along with elevated HO‐1 expression (Figure 3D), corroborating the antioxidant and anti‐inflammatory effects of f‐CA(OH).

Following validation of the potent ROS‐scavenging activity of f‐CA(OH), the regulatory effects of f‐CA(OH) and f‐CA(OH)‐laden exosomes on macrophage oxidative stress were further evaluated by assessing mitochondrial membrane potential via JC‐1 staining (Figure 3E; Figure S17, Supporting Information). LPS treatment reduced red fluorescence intensity (74.39) and increased green fluorescence intensity (79.80), which suggests mitochondrial depolarization and potential cellular harm. In contrast, both the LPS+fCA and LPS+fCA‐BExo groups exhibited higher red fluorescence intensities (81.24 and 82.47, respectively) compared to the LPS group, although still below to control levels (84.41), suggesting that fCA partially mitigates LPS‐induced damage, with exosomes providing further protective benefits.

Flow cytometric analysis (Figure 3F) demonstrated that LPS stimulation induced a significant shift toward pro‐inflammatory M1 polarization (CD86⁺ macrophages: 28.64%), which was substantially attenuated by both f‐CA(OH) monotherapy (18.50%) and f‐CA(OH)‐loaded exosome treatment (14.80%). Notably, the combinatorial regimen showed superior efficacy in promoting immunoregulatory M2 polarization (CD206⁺ macrophages: 77.71%, Figure 3G). Western blot analysis (Figure 3H) corroborated these findings, demonstrating that the f‐CA(OH)‐exosome combination significantly upregulated CD206 expression while suppressing CD86 expression, thereby confirming the effective promotion of an immune regulatory M2 phenotype. To evaluate functional macrophage changes, ELISA was employed to measure the secretion levels of the pro‐inflammatory cytokine TNF‐α and the immunoregulatory cytokine TGF‐β. The data demonstrated that the synergistic action of f‐CA(OH)‐laden exosomes decreased TNF‐α secretion by 69.1% while increasing TGF‐β secretion approximately twofold (Figures S18, S19, Supporting Information). This reprogramming of the immune microenvironment establishes a key regulatory mechanism for suppressing excessive osteoclast activation.

In summary, f‐CA(OH) effectively mitigates oxidative damage through ROS scavenging and Nrf2/HO‐1 pathway activation, while its synergistic application with customized exosomes reprograms macrophage polarization by inhibiting the pro‐inflammatory M1 phenotype and enhancing the immunoregulatory M2 phenotype, thereby ameliorating the inflammatory microenvironment associated with osteoporosis.

### 2.5 Engineered Exosomes Selectively Target and Block the PI3K/AKT Pathway to Suppress Osteoclast Precursor Maturation

To elucidate the molecular mechanisms by which exosomes regulate osteoclast differentiation, we established a RANKL‐induced RAW264.7 osteoclastogenesis model and systematically assessed the spatiotemporal regulation of the PI3K/AKT signaling by f‐CA(OH) nanomaterials, stem cell‐derived exosomes, and engineered exosomes (fCA‐BExo) under inflammatory conditions. Following five days of induction, tartrate‐resistant acid phosphatase (TRAP) staining revealed that LPS stimulation, mimicking an inflammatory microenvironment, significantly induced the formation of multinucleated macrophage fusion and elevated TRAP activity to 3.2‐fold that of the control (Figure S20, Supporting Information). Treatment with f‐CA(OH) alone partially attenuated the LPS‐induced inflammatory response, decreasing cell fusion and multinucleated osteoclasts formation. Notably, both stem cell exosomes and engineered exosomes exhibited a more pronounced inhibitory effect. Quantitative analysis of TRAP activity in the culture supernatant was consistent with the staining results, with the fCA‐BExo group exhibiting only 52.1% of the TRAP activity observed in the LPS group, significantly outperforming the Exo group at 72.3% (Figure S21, Supporting Information), thereby demonstrating that the engineered modifications endow exosomes with superior anti‐osteoclastogenic properties.

Moreover, immunofluorescence imaging on day 3 of induction revealed dynamic changes in PI3K/AKT signaling. LPS stimulation induced characteristic activation patterns, with phosphorylated AKT (red) distributed throughout the perinuclear cytoplasm and plasma membrane, accompanied by elevated levels of phosphorylated PI3K (green) (Figure 3J). In contrast, fCA‐BExo treatment caused intracellular redistribution of p‐AKT and reduced activation levels (Figure S22, Supporting Information), suggesting that engineered exosomes modulate signal transduction by altering PI3K spatial activity. These findings were corroborated in THP‐1 cells, which showed similar patterns of PI3K/AKT modulation (Figure 3J). Quantitative Western blot analysis (Figure 3I) confirmed significant reduction of p‐AKT expression in the fCA‐BExo group compared to LPS controls, consistent with the observed changes in perinuclear p‐AKT signals. Collectively, these results demonstrate that under inflammatory conditions, customized exosomes inhibit PI3K/AKT signaling, thereby effectively disrupting RANKL‐induced osteoclast maturation and TRAP secretion.

### 2.6 f‑CA(OH) Co‑Culture Enhances the Secretion of Stem Cell Exosomes and Modulates Their BMP2 Content

**Figure 4:**
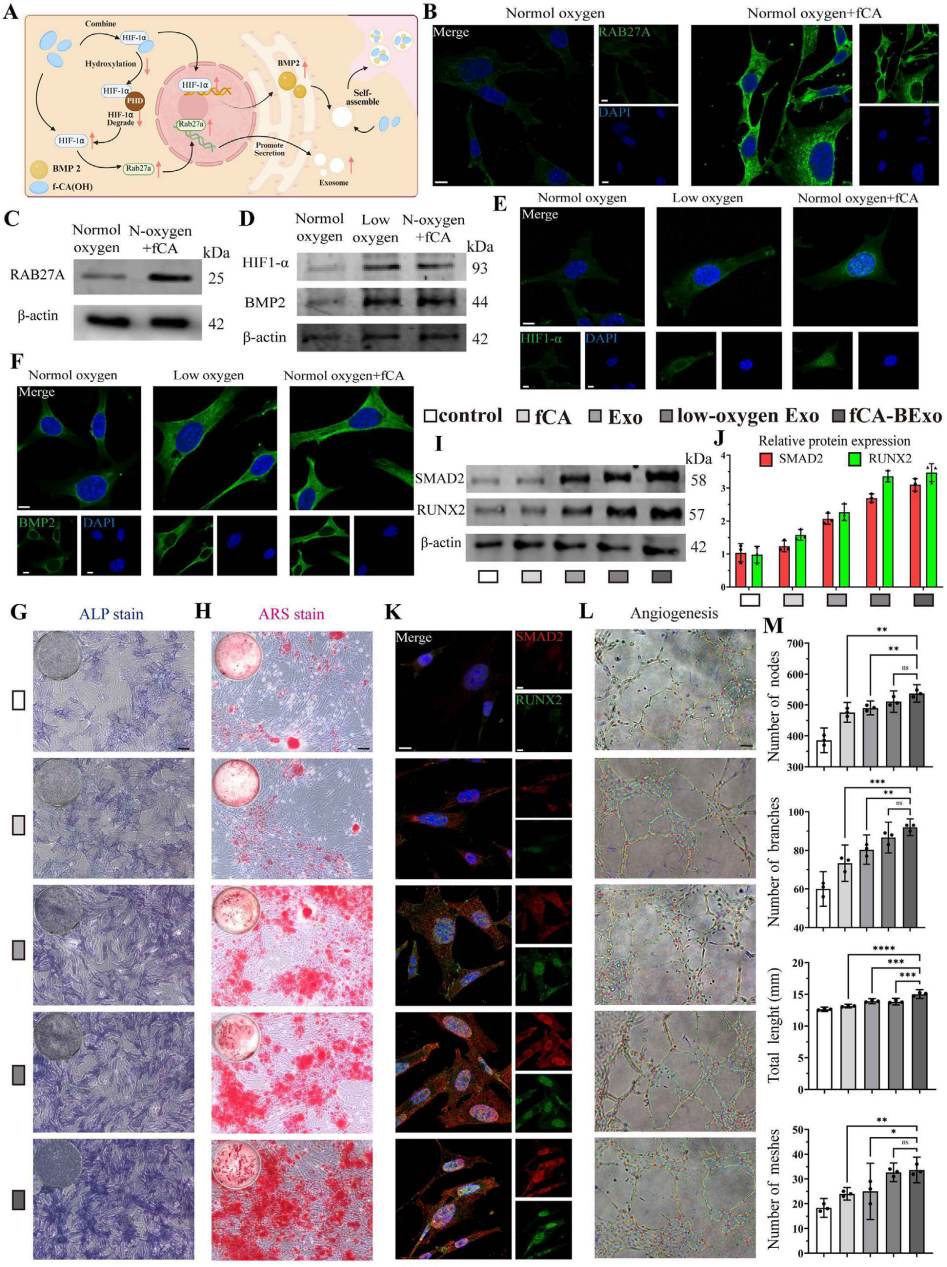
f‐CA(OH) enhances exosome secretion and osteogenesis. A) A schematic diagram depicting the metabolic biosynthesis and self‐assembly processes mediated by f‐CA(OH)‐BExo in BMSCs (Created using Biorender). B) Confocal microscopy images that reveal the immunofluorescence expression of RAB27A in BMSC cells that underwent co‐culture with f‐CA(OH) (scale bar: 10 µm). C) Western blot assessment of RAB27A protein levels in BMSC cells following co‐culture with f‐CA(OH). D) Western blot evaluation of HIF1‐α and BMP2 protein expression in BMSC cells after being co‐cultured with f‐CA(OH). E, F) Confocal laser scanning microscopy (CLSM) images illustrating the subcellular localization and immunofluorescence profiles of HIF1‐α and BMP2 in BMSC cells co‐cultured with f‐CA(OH) (scale bar: 10 µm). G) Alkaline phosphatase (ALP) staining results for BMSC cells following 7 days of culture under varied conditions. H) Alizarin red S (ARS) staining results for BMSC cells after 14 days of culture under different settings (scale bar: 100 µm). I) Investigation through Western blot of SMAD2 and RUNX2 protein expression levels in BMSC cells under diverse conditions. J) Quantitative assessment of SMAD2 and RUNX2 protein levels determined by Western blot grayscale analysis (n = 3). K) CLSM images displaying the subcellular localization and immunofluorescence expression of SMAD2 and RUNX2 in BMSC cells (scale bar: 10 µm). L) Tube formation assay of HUVECs cultured under different conditions for 2 days (scale bar: 200 µm). M) Quantitative analysis of angiogenesis capacity across treatment groups (n = 3). All results are shown as mean ± SD. *p < 0.05, **p < 0.01, ***p < 0.001, ****p < 0.0001.

Our study demonstrates that f‐CA(OH) co‐culture significantly enhances the release of extracellular vesicle (EV) from mesenchymal stem cells (MSCs) (Figure S23, Supporting Information) while modulating their BMP2 content (Figure **4D**). To investigate the dynamics of EV secretion, we examined the expression of the key regulatory factor RAB27a. Immunofluorescence quantification revealed 2.1‐fold higher RAB27a intensity in the f‐CA(OH)‐treated group compared to the control (Figure 4B), while Western blot analysis further confirmed a 2.3‐fold upregulation in protein expression (Figure 4C; Figure S24, Supporting Information). These results suggest that f‐CA(OH), owing to its oxygen‐vacancy–rich peroxidase‐mimetic activity, promotes extracellular vesicle (EV) release by decomposing hydrogen peroxide to generate oxygen,^[^ ^45^, ^46^, ^47^ ^]^ thereby raising local oxygen tension (Figure S25, Supporting Information) and intracellular ATP levels, which activate RAB27a and trigger the RAB27a‐dependent secretory pathway.

Mechanistically, we found that f‐CA(OH) continuously releases cobalt ions (Co^2^⁺) under physiological conditions, inhibiting prolyl hydroxylases (PHDs) and blocking oxygen‐dependent HIF1‐α degradation to mimic hypoxia signaling. Western blot analysis revealed a significant 1.9‐fold increase in HIF1‐α protein expression in the f‐CA(OH)‐treated group compared to normoxic conditions (Figure 4D; Figure S26, Supporting Information), reaching levels comparable to those observed in the hypoxia group (5% O_2_). Immunofluorescence co‐localization analysis further confirmed nuclear accumulation of HIF1‐α in f‐CA(OH)‐treated cells, forming characteristic nuclear foci similar to those in the hypoxia group (Figure 4E; Figure S27, Supporting Information). Moreover, nuclear interaction between HIF1‐α and HIF1‐β directly activated BMP2 gene transcription (Figure 4F), leading to a marked upregulation of BMP2 protein expression, reaching 2.2‐fold and 2.0‐fold of normoxic levels in the hypoxia and f‐CA(OH) groups, respectively (Figure S26, Supporting Information). To mechanistically validate this pathway, pharmacological inhibition of HIF1‐α with 2‐methoxyestradiol (2‐ME2) completely abrogated the f‐CA(OH)‐mediated upregulation of BMP2 (Figure S28, Supporting Information), conclusively demonstrating that f‐CA(OH) enhances BMP2 expression through HIF1‐α stabilization.

### 2.7 Engineered Exosomes Enhance the Osteogenesis of Pre‐Osteoblasts through the SMAD2/RUNX2 Pathway

Given the inherent extracellular matrix‐targeting properties of stem cell‐derived exosomes and their endogenous osteogenic protein cargo, this study employed f‐CA(OH) nanomaterials to engineer functionalized exosomes (fCA‐BExo) and systematically elucidate their multidimensional regulatory network in promoting osteogenic differentiation of BMSCs.

To explore their possible involvement in bone formation, we enriched osteogenic differentiation media with f‐CA(OH) nanomaterials, exosomes produced under varying culture conditions, and exosomes specifically prompted by f‐CA(OH). Early osteogenic differentiation was evaluated by alkaline phosphatase (ALP) staining (Figure 4G). In comparison to the PBS control, there was a modest increase in ALP expression within the f‐CA(OH)‐treated cohort, while all groups receiving exosome treatment demonstrated a marked enhancement in ALP activity. Remarkably, the tailored fCA‐BExo group exhibited the most significant effect, with the intensity of ALP staining greatly exceeding that of the f‐CA(OH) only group, highlighting a robust synergistic osteogenic impact between the engineered exosomes and the nanomaterial.

To assess the long‐term capacity for mineralization, staining with alizarin red S (ARS) was conducted (Figure 4H). Following a 14‐day culture period, calcium deposition was observed in the f‐CA(OH) group; however, this increase was not significantly different when compared to the control group. In contrast, groups treated with exosomes demonstrated a pronounced enhancement in the formation of calcium nodules, with the fCA‐BExo group exhibiting the greatest increase in mineral deposition when compared to both the general exosome group and the hypoxia‐induced exosome group. These results indicate that fCA‐BExo has a superior potential for osteogenic mineralization.

To elucidate the underlying molecular mechanisms, we analyzed key osteogenic signaling pathways. Western blot analysis (Figure 4I, J) revealed that all treatment groups exhibited varying degrees of upregulation in SMAD2, a pivotal osteogenic signaling molecule, and RUNX2, a master osteogenic transcription factor. The fCA‐BExo group displayed the highest expression levels, with SMAD2 and RUNX2 levels increasing by 2.1‐fold and 2.5‐fold, respectively, in comparison to the control. Concurrently, qRT‐PCR analysis confirmed elevated SMAD2 and RUNX2 gene expression levels in treated cells (Figure S29, Supporting Information). Furthermore, immunofluorescence co‐localization analysis (Figure 4K) demonstrated significantly enhanced co‐localization signals of SMAD2 (red fluorescence) and RUNX2 (green fluorescence) in the fCA‐BExo group (Figure S30, Supporting Information), with a marked increase in nuclear accumulation of RUNX2, far exceeding that observed in the normal control group. These findings imply that fCA‐BExo facilitates osteogenic differentiation primarily by activating the SMAD2/RUNX2 signaling axis, thereby driving the transcription of osteogenesis‐related genes. Consistent with these results, parallel immunofluorescence staining in MC3T3‐E1 cells demonstrated similar patterns of SMAD2/RUNX2 activation (Figure S31, Supporting Information).

In conclusion, we successfully engineered customized exosomes (fCA‐BExo) via f‐CA(OH) nanomaterial induction. These exosomes not only retained the natural homing properties of native exosomes but also exhibited significantly enhanced osteogenic differentiation capacity. Mechanistically, their efficacy was primarily attributed to the synergistic regulation of SMAD2 phosphorylation and RUNX2 nuclear translocation, ultimately amplifying the osteogenic potential of BMSCs.

Given the well‐established role of HIF1‐α activation in neovascularization within osteoporotic microenvironments, we investigated whether our engineered exosomes could mitigate osteoporosis through angiogenic modulation. Using the customized fCA‐BExo to induce vasculogenic differentiation of endothelial cells, we observed that all tested components (f‐CA(OH) nanoparticles, plain exosomes, and fCA‐BExo) enhanced angiogenesis (Figure 4L). Notably, fCA‐BExo demonstrated superior pro‐angiogenic effects, significantly increasing tubular network complexity as evidenced by elevated node number, branch points, total length, and mesh formation (Figure 4M) compared to controls.

### 2.8 In Vivo Bone Marrow Targeting Evaluation and Biosafety Assessment

**Figure 5:**
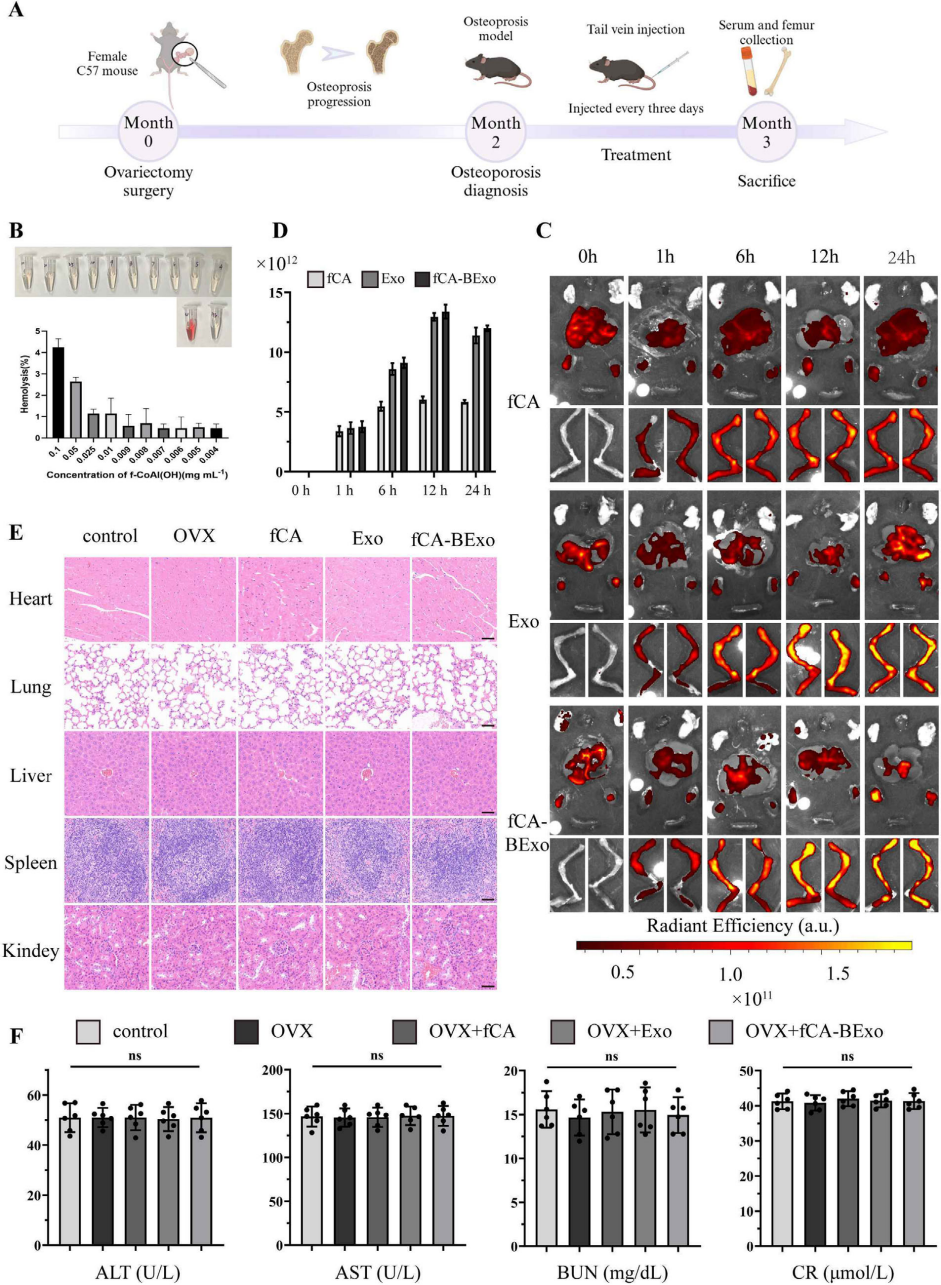
Engineered exosomes target bone marrow with systemic biosafety in OVX mice. A) Schematic diagram of osteoporotic mouse model establishment and experimental treatment protocol (By Biorender). B) Hemolysis rate analysis of f‐CA(OH) at different concentrations in vitro (safety threshold: < 5%). C) In vivo fluorescence imaging of drug distribution in mice at 0, 1, 6, 12, and 24 h post‐tail vein injection (scale bar: whole‐body imaging). D) Quantitative analysis of fluorescence intensity in bone marrow cavities over time (n = 6). E) Hematoxylin and eosin (HE) staining results for the heart, lung, liver, spleen, and kidney tissues collected 24 h after treatment (scale bar: 50 µm). F) Measurement of serum levels for blood alanine aminotransferase (ALT), aspartate aminotransferase (AST), urea nitrogen (BUN) and creatinine (CR) to evaluate hepatic and renal metabolic functions (n = 6). All results are shown as mean ± SD.

Given that the engineered exosomes not only suppressed inflammatory responses but also promoted the osteogenic differentiation of mesenchymal stem cells in vitro, we then explored their potential application for osteoporosis treatment in vivo. After conducting bilateral ovariectomy on C57 mice to create an osteoporotic model, 30 mice were randomly divided into five distinct groups: a control group (sham‐operated), a PBS group, an f‐CA(OH) group, an exosome (Exo) group, and a tailored exosome (fCA‐BExo) group. Once it was established that each formulation fell within a biologically safe range (hemolysis rate < 5%; **Figure** **5**B), 150 µL of each treatment (100 µg mL^−1^) was intravenously administered via tail vein injection. In vivo fluorescence imaging at 0, 1, 6, 12, and 24 h post‐injection provided preliminary assessment of treatment distribution (Figure 5C).

The results indicated that the fluorescence signal in the bone marrow cavity progressively increased from 0 to 12 h post‐injection, plateaued at 12 h, and then slightly decreased between 12 and 24 h (Figure 5D). Notably, groups containing exosomes demonstrated a more significant accumulation in the bone marrow cavity after 6 h compared to the group receiving only f‐CA(OH). Additionally, enhanced fluorescence signals were observed in the liver and kidney regions across all groups (Figure 5C), suggesting that the drugs were primarily metabolized via the hepatic and renal pathways. High‐resolution cellular imaging of isolated marrow cells revealed efficient uptake of DiO‐labeled exosomes and subsequent release of encapsulated nanosheets, with the fCA‐BExo group showing markedly enhanced f‐CA(OH) accumulation compared to f‐CA(OH) control (Figure S32, Supporting Information). These results visually confirm that exosomal encapsulation significantly improves targeted delivery of f‐CA(OH) to bone marrow.

To explore the effects of the drugs on key organs, mice were euthanized 24 h after injection, and heart, lung, liver, spleen, and kidney tissues were harvested for HE staining (Figure 5E). Histological assessments indicated that there were no notable pathological changes in any of the tissues when compared to the control group. These findings were further corroborated by long‐term toxicity studies extending to 30, 60, and 90 days, which demonstrated maintained histological integrity across all examined organs (Figure S33, Supporting Information). Additionally, biochemical evaluations of blood samples taken at the 24‐hour mark revealed that both hepatic and renal metabolic parameters stayed within their normal limits (Figure 5F), thereby reinforcing the favorable biosafety profiles of the formulations.

### 2.9 Evaluation of the Therapeutic Efficacy of Engineered Exosomes

**Figure 6:**
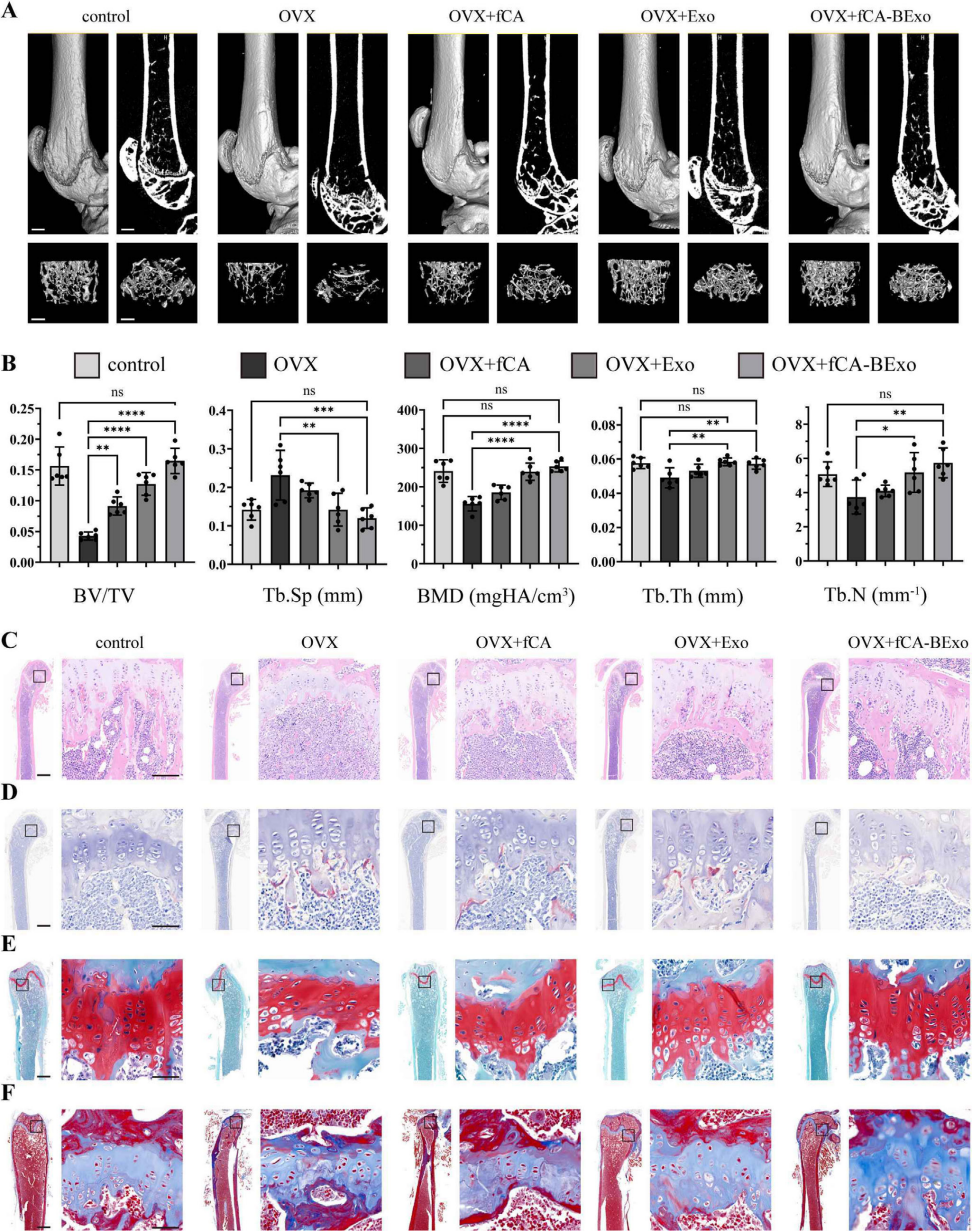
Engineered exosomes reverse bone loss and restore microarchitecture in vivo. A) 3D micro‐CT reconstructions and a comparison of the trabecular microarchitecture in femurs from different treatment groups (scale bar: 0.5 mm). B) Quantitative measurements of bone volume fraction (BV/TV), trabecular separation (Tb.Sp), bone mineral density (BMD), trabecular thickness (Tb.Th), and trabecular count (Tb.N) (n = 6). C) Hematoxylin and eosin (HE) staining showing trabecular structure and metaphyseal form in femurs from different treatment groups (scale bar: 100 µm). D) Tartrate‐resistant acid phosphatase (TRAP) staining utilized for quantitative assessment of osteoclast quantity and function (scale bar: 50 µm). E) Safranin O/fast green staining revealing trabecular mineralization and morphology of subchondral bone (scale bar: 50 µm). F) Collagen fiber content and mineralization assessment in bone matrix through Masson's trichrome staining (scale bar: 50 µm). All results are shown as mean ± SD. ns ≥0.05, *p < 0.05, **p < 0.01, ***p < 0.001, ****p < 0.0001.

In this study, after 30 days of treatment, micro‑CT scans revealed that the engineered exosome group exhibited a marked improvement in the trabecular architecture of the femur compared to the OVX+PBS group (**Figure** **6**A). Quantitative analysis revealed significant therapeutic effects, with increased bone volume fraction (BV/TV) and bone mineral density (BMD) indicating enhanced mineralization, along with elevated trabecular number (Tb.N) and reduced trabecular separation (Tb.Sp) (Figure 6B). These structural improvements demonstrate effective attenuation of bone resorption and osteoporotic bone loss.

Hematoxylin and eosin (HE) staining (Figure 6C) demonstrated that the trabecular structure and metaphyseal morphology in the fCA‑BExo group were substantially restored relative to the OVX group, with bone matrix tissue exhibiting a dense and continuous arrangement, further confirming the improvement in bone integrity. Concurrently, TRAP staining (Figure 6D) showed that engineered exosome treatment significantly reduced both the number and activity of osteoclasts, indicating a robust inhibitory effect on bone resorption. Safranin O/fast green staining (Figure 6E) revealed improvements in trabecular architecture and mineralization, with the subchondral bone morphology approaching normalcy, while Masson staining (Figure 6F; Figure S34, Supporting Information) further verified that the fCA‐BExo group exhibited significantly greater collagen fiber deposition compared to OVX controls. Collectively, these results demonstrate that engineered exosomes effectively restore bone microarchitecture while establishing a physiological equilibrium between osteogenesis and bone resorption through dual modulation of osteoclast inhibition and bone matrix deposition, providing experimental evidence for the treatment of osteoporosis.

### 2.10 The Regulatory Role of Engineered Exosomes in the Osteoporotic Bone Marrow Microenvironment

**Figure 7:**
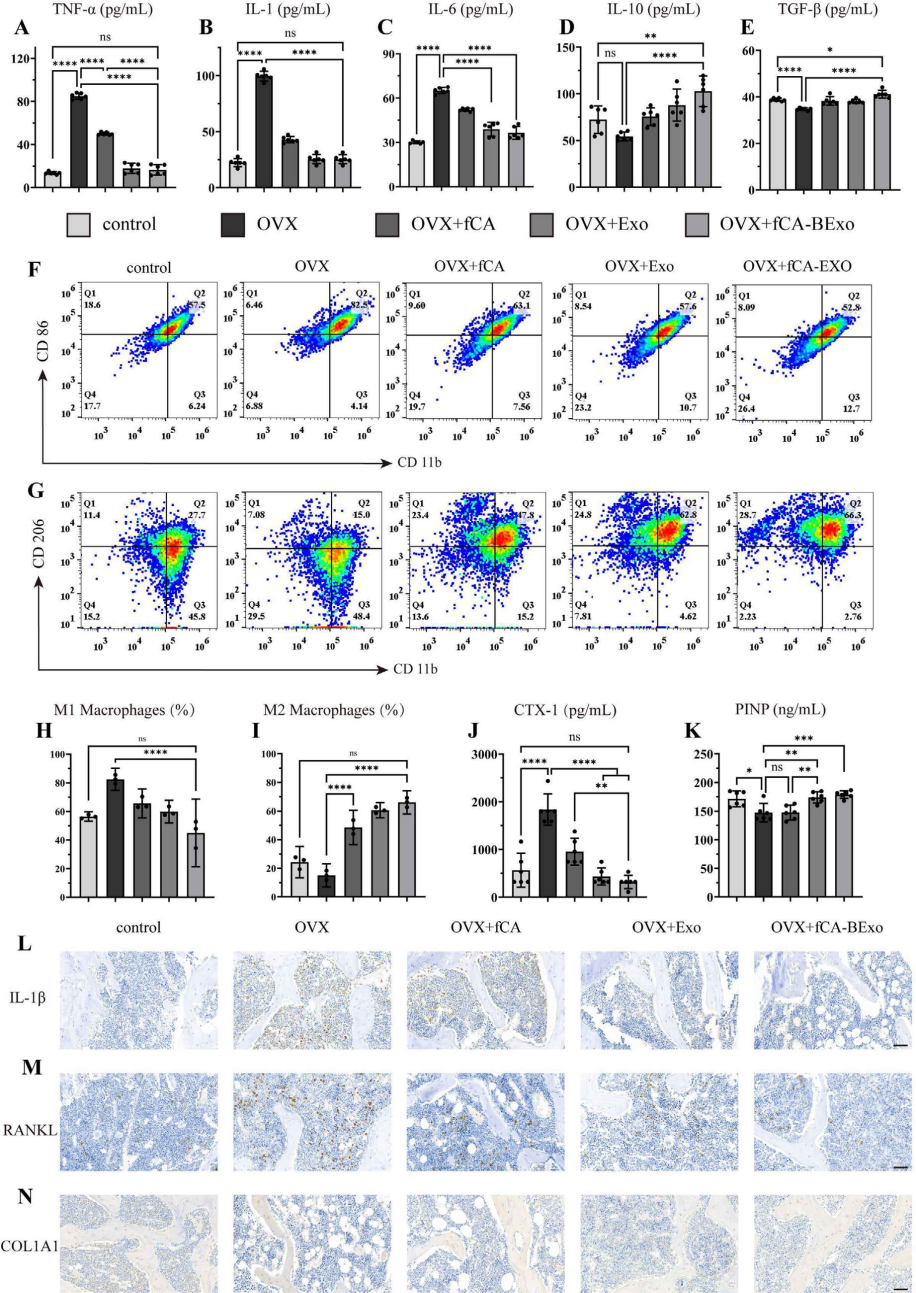
Engineered exosomes reprogram immune microenvironment and bone remodeling in osteoporosis. A) Analysis of TNF‐α levels in bone marrow supernatants from femurs of mice across treatment groups (n = 6). B) IL‐1β levels in bone marrow supernatants evaluated through ELISA (n = 6). C) ELISA analysis of IL‐6 levels in bone marrow supernatants (n = 6). D) ELISA analysis of anti‐inflammatory cytokine IL‐10 levels in bone marrow supernatants (n = 6). E) ELISA analysis of reparative cytokine TGF‐β levels in bone marrow supernatants (n = 6). F) Flow cytometry analysis of M1 macrophage (CD11b⁺CD86⁺) proportions in bone marrow. G) Flow cytometry analysis of M2 macrophage (CD11b⁺CD206⁺) proportions in bone marrow. H) Quantitative assessment of M1 macrophage proportions (n = 3). I) Quantitative evaluation of M2 macrophage proportions (n = 3). J) ELISA analysis of osteoclast activity marker CTX‐1 levels in bone marrow supernatants (n = 6). K) ELISA analysis of osteogenic marker PINP levels in bone marrow supernatants (n = 6). L) Immunohistochemical (IHC) staining of IL‐1β expression in bone marrow (scale bar: 50 µm). M) IHC staining of RANKL expression (osteoclast differentiation marker, scale bar: 50 µm). N) IHC staining of COL1A1 expression (osteoblast differentiation marker, scale bar: 50 µm). All results are shown as mean ± SD.ns ≥0.05, *p < 0.05, **p < 0.01, ***p < 0.001, ****p < 0.0001.

Based on the identified bone‐protective properties, we conducted a deeper analysis into the mechanistic basis of fCA‐BExo's modulation of the osteoporotic inflammatory microenvironment. After euthanization, femoral bone marrow was collected and supernatant cytokine levels were analyzed by ELISA. In comparison to the OVX+PBS group, all treatment groups demonstrated a significant alteration in cytokine secretion profiles: levels of pro‐inflammatory cytokines (IL‐1β, IL‐6, and TNF‐α) were markedly reduced in the fCA‑BExo cohort (**Figure** **7**A–C), while both the anti‐inflammatory cytokine IL‐10 (Figure 7D) and the reparative cytokine TGF‑β (Figure 7E) alongside increased. These findings suggest that the engineered exosomes efficiently inhibit local inflammation, thus contributing to the improvement of osteoporotic conditions.

Flow cytometric analysis further revealed that fCA‑BExo substantially improved the immune cell composition within the bone marrow microenvironment. Specifically, the proportion of M1 macrophages (CD11b⁺CD86⁺) was reduced to 54.8% of that observed in the OVX group (Figure 7F, H), while the proportion of M2 macrophages (CD11b⁺CD206⁺) increased by 4.4‐fold relative to controls (Figure 7G, I), demonstrating superior immunomodulatory effects compared to other treatment groups.

Immunohistochemical analysis (Figure 7L; Figure S35, Supporting Information) revealed minimal expression of pro‐inflammatory cytokines (IL‐1β, IL‐6, TNF‐α) in healthy bone, with marked elevation in OVX mice. Treatment with nanomaterials and engineered exosomes – particularly fCA‐BExo restored these cytokines to near‐normal levels while significantly increasing anti‐inflammatory IL‐10 (Figure S36, Supporting Information). The semi‐quantitative analysis of immunohistochemical images (Figure S37, Supporting Information), consistent with ELISA results, demonstrates that engineered exosomes possess the dual capability of remodeling inflammatory microenvironments and regulating macrophage polarization.

At the same time, the results from ELISA indicated that the CTX level, which serves as a marker for osteoclast activity, showed a substantial decrease in the fCA‑BExo group (Figure 7J). Meanwhile, PINP, an indicator of osteogenesis, revealed a notable increase (Figure 7K). Further validation through immunohistochemical staining supported these results: the application of engineered exosomes led to a significant rise in the number of COL1A1‐positive cells and a considerable decrease in RANKL expression (Figure 7M, N; Figure S37, Supporting Information). This suggests an enhancement of osteoblastic activity alongside a reduction in osteoclastic function, consistent with the previously noted micro‑CT and histological observations. Collectively, these results demonstrate that engineered exosomes modulate the osteoporotic bone marrow microenvironment through multi‐target interventions, regulating cytokine release and macrophage polarization to promote osteogenesis while inhibiting osteoclastogenesis, thus providing compelling experimental evidence for their potential in the treatment of osteoporosis.

Consistent with the in vitro findings, Immunofluorescence analysis of femoral sections demonstrated that p‐Akt fluorescence intensity in bone marrow cells was significantly elevated in the OVX group. This activation was suppressed by all three treatments (Figure S38, Supporting Information). The superior inhibitory effect of fCA‐BExo corroborates our in vitro findings that the engineered exosome synergistically targets PI3K‐AKT signaling in osteoclasts.

## 3 Conclusion

Based on transcriptomic analyses, we identified significant dysregulation of immune‐related genes in the bone marrow of osteoporotic mice, accompanied by aberrant activation of inflammatory chemokines and the PI3K/AKT pathway, implicating an altered immune microenvironment as a key factor in bone loss. To address these abnormalities, our team successfully synthesized f‑CA(OH) nanosheets with enzyme‐mimetic activity that effectively scavenge reactive oxygen species (ROS) via activation of the Nrf2/HO‑1 pathway, thereby markedly improving the local immune milieu. Moreover, by co‐culturing f‑CA(OH) with mesenchymal stem cells, exploiting its ability to release Co^2^⁺ to mimic hypoxic signals and upregulate BMP2 expression, we engineered customized exosomes (fCA‑BExo) that combine metal enzyme functionality with a high concentration of osteoinductive proteins. In vitro, fCA‑BExo exert synergistic effects through dual mechanisms: they inhibit osteoclast differentiation by blocking PI3K/AKT signaling and promote osteogenic differentiation and mineralization by activating the SMAD2/RUNX2 axis. In vivo studies further demonstrated that fCA‑BExo preferentially accumulate in bone tissue, significantly improve trabecular architecture in OVX mice, and exhibit no evident hepatotoxicity or nephrotoxicity, underscoring their potential for the treatment of osteoporosis.

## Author Contributions

R.T., G.L., C.W., and M.C. contributed equally to this research work. F.G., M.G., and H.C. conceived and designed the study. R.T. and G.L. synthesized the materials, carried out the structural characterizations, and performed catalytic experiments. C.W. and M.C. performed the in vitro study and in vivo studies. G.M. and P.W. assisted in conducting in vitro and in vivo studies. S.D. assisted with the catalytic experiments. J.L., Y.L., C.J. and G.W. analyzed the results and drafted the manuscript. All authors participated in discussing the results and provided feedback on the manuscript.

## Conflict of Interest

The authors declare no conflict of interest.

## Supporting information



Supporting Information

## Data Availability

The data that support the findings of this study are available from the corresponding author upon reasonable request.
