# Supplementary material for: Transcriptomics Guided Engineering of Exosome‐Encapsulated Bifunctional Nanosheets Targeting the Immune‐PI3K/Akt Axis for Osteoporosis Therapy
Source: Adv Sci (Weinh). 2025 Sep 29;12(47):e11327. doi: 10.1002/advs.202511327 (PMC12713082; doi:10.1002/advs.202511327)
Supplement: Supplementary file 1 — Supporting Information [file ADVS-12-e11327-s001.docx]

Supporting Information

**Transcriptomics Guided Engineering of Exosome-Encapsulated Bifunctional Nanosheets Targeting the Immune-PI3K/Akt Axis for Osteoporosis Therapy**

Rongze Tang, Guanhong Liu, Congcong Wang, Minghao Chao, Guangyu Ma, Pengjin Wei, Siling Du, Junjie Li, Yufan liu, Chaonan Jing, Guoquan Wu, Ming Guan*, Hongliang Chen*, and Fenglei Gao*

Rongze Tang, Guanhong Liu, Pengjin Wei, Guoquan Wu, Hongliang Chen, Fenglei Gao

Department of Orthopedics,

The Affiliated Hospital of Xuzhou Medical University,

Jiangsu 221004, China.

Email: xzchenhl@sina.com (H. Chen), flgao@xzhmu.edu.cn (F. Gao).

Rongze Tang, Guanhong Liu, Guangyu Ma, Pengjin Wei, Siling Du, Junjie Li, Yufan liu, Chaonan Jing, Guoquan Wu, Fenglei Gao

Key Laboratory of New Drug Research and Clinical Pharmacy,

Xuzhou Medical University,

Jiangsu 221004, China.

Email: flgao@xzhmu.edu.cn (F. Gao)

Ming Guan, Fenglei Gao

Department of Laboratory Medicine,

Huashan Hospital, Shanghai Medical College,

Fudan University,

Shanghai 200040, China.

Email: [guanming88@yahoo.com](mailto:guanming88@yahoo.com) (M. Guan), flgao@xzhmu.edu.cn (F. Gao).

Congcong Wang

Department of Orthopedics,

The Affiliated Huai'an Hospital of Xuzhou Medical University,

The Second People's Hospital of Huai'an,

Huai'an 223002, China.

Minghao Chao

Department of Orthopaedic Surgery,

Lishui Central Hospital and Fifth Affiliated Hospital of Wenzhou Medical University,

Lishui, 323000, China.

**Experimental Section**

*Materials*: All chemicals utilized in the experiments were of analytical grade and required no additional purification. Cobalt (II) nitrate hexahydrate (Co(NO_3_)_2_·6H_2_O), aluminum nitrate nonahydrate (Al(NO_3_)_3_·9H_2_O), formamide (CH_3_NO), sodium hydroxide (NaOH), and ethanol (CH_3_CH_2_OH) were purchased from Aladdin Reagent. DMEM medium and fetal bovine serum (FBS) were obtained from Gibco (Shanghai, China). CCK-8 assay kit and dexamethasone were procured from TargetMol (USA). 2', 7'-Dichlorodihydrofluorescein diacetate (DCFH-DA) was supplied by Sigma-Aldrich (Shanghai, China). Fluorescein isothiocyanate (FITC) was acquired from MedChemExpress (China). Cell Viability Assay Kit, JC-1 Apoptosis Detection Kit, and Apoptosis Detection Kit were purchased from KeyGEN (Nanjing, China). Protease inhibitor, phosphatase inhibitor, and Alizarin Red S staining solution (2 %, pH 4.2) were sourced from Beyotime Biotechnology (Shanghai, China). PAGE Gel Quick Preparation Kit (8 %, 10 %) and Alkaline Phosphatase Stain Kit were provided by YEASEN Biotechnology (Shanghai, China). Polyclonal antibodies against Cathepsin K, PI3K, RAB27A, BMP2, SMAD2, and RUNX2, as well as ELISA kits for TNF-α, IL-1β, IL-6, IL-10, and TGF-β1, were purchased from Proteintech (Wuhan, China). Antibodies targeting p-AKT, HIF1-α, and COL1A1 were obtained from Thermo Fisher Scientific (USA). RAW 264.7 (RRID: CVCL_0493), BMSC (RRID:CVCL_VG66), MC3T3-E1 (RRID:CVCL_0409) and THP-1 (RRID：CVCL_0006） cell lines were provided by HyCyte (China).

*Instrumentation*: Exosomes were collected using Optima XPN-100 centrifugation. The morphology and structure of as-prepared f-CA(OH)/fCA-BExo were characterized using transmission electron microscopy imaging (FEI Tecnai G2 Spirit Twin, Holland). Scanning transmission electron microscopy (STEM) and elemental mapping images were obtained to the elemental composition. The crystal structure of the sample was determined by X-ray diffractometer (Bruker D8 Advance) with Cu Kα radiation. UV-vis absorption spectra of different samples were recorded by an Evolution 220 UV-Visible Spectrophotometer (Thermo Fisher Scientific). All fluorescence measurements were performed on a Hitachi FL4600 fluorescence spectrophotometer (Kyoto, Japan). Fluorescent images were captured using a laser scanning confocal microscope (Leica STELLARlS 5, Germany). DLS and Zeta potential analysis were performed at room temperature on a Zetasizer (Nano-Z, 60 Malvern, UK). Flow cytometric analysis was performed on a FACS Calibur flow cytometer (Becton Dickinson, USA). CCK-8 assay was performed using a microplate reader (Thermo Fisher Scientific) at 450 nm. In vivo fluorescence imaging was recorded on LB983 Night OWL II* Imaging System. (Berthold, Germany). The photothermal images were recorded by a FLUKE infrared (IR) thermal camera. The 660 nm laser was emitted from an LWIRL660-10W-F fiber-coupled laser.

*Animals*: For animal model establishment, female C57BL/6J mice, aged six weeks, were procured from GemPharmatech Co., Ltd. The mice were housed under SPF conditions at Xuzhou Medical University with sterilized food and water provided ad libitum. The ethical approval for all animal-related experiments was granted by the Ethics Committee of Xuzhou Medical University, under the approval number 202403T014.

*Synthesis and Characterization of f-CA(OH)*: A solution labeled as A was created by dissolving 218.275 mg of cobalt (II) nitrate hexahydrate (Co(NO_3_)_2_·6H_2_O) in 20 mL of deionized water, producing a red-hued solution. In a separate procedure, a total of 93.783 mg of aluminum nitrate nonahydrate (Al(NO_3_)_3_·9H_2_O) was combined with 30 mL of deionized water designated as B. Both solutions A and B were then added dropwise over a period of 10 min to 20 mL of a preheated (85 °C) 23% formamide solution while being stirred vigorously, ensuring that the reaction maintained a pH of 10. Under continuous magnetic stirring at 200 rpm, the mixture slowly changed into a pale red, turbid suspension. The resulting precipitate was then gathered through centrifugation at 8000 rpm for 10 min, following sequential washes with ethanol and deionized water (twice for each), leading to the formation of f-CA(OH) nanosheets.

*Cell Proliferation and Cytotoxicity Assay*: BMSCs and RAW 264.7 cells were seeded in 96-well plates at a density of 10,000 cells/well and treated with f-CA(OH) solutions at varying concentrations (0.1–1.0 mg/mL) for 12 h. Subsequently, CCK-8 working solution (10 % CCK-8 reagent in 90 % DMEM medium) was added to each well. After incubating for 2 h at 37 °C, the absorbance was measured at 450 nm using a microplate reader to evaluate the effects of f-CA(OH) on cell proliferation and cytotoxicity.

*Extraction of fCA-BExo*: BMSC cells were grown in 6-well plates or 35 mm diameter cell culture dishes at a density of 3×10^6^ cells per well. Upon reaching approximately 60% confluency, the cells were exposed to a complete culture medium supplemented with 0.1 mg/mL of f-CA(OH) for a duration of 12 h. Following this, the complete culture medium was substituted with a serum-free version that maintained the equivalent concentration of f-CA(OH), and the cells were incubated for another 12 h, resulting in a confluency greater than 90%.The culture supernatant was gathered and underwent a series of centrifugation steps: first at 300×g for 10 min to eliminate cell debris, then set 1990×g for 30 min taking the supernatant, and finally, ultracentrifugation was conducted at 169,000×g (4 °C, for 120 min) to concentrate the exosomes. The resulting pellet was then resuspended in PBS, aliquoted into smaller volumes, and stored at -80 °C.

*Characterization of fCA-BExo*: Physicochemical Characterization: A total of five milligrams of lyophilized fCA-BExo powder was dissolved in 10 mL of phosphate-buffered saline. The particle size distribution and Zeta potential of fCA-BExo were evaluated using a nanoparticle tracking analyzer (NTA). Furthermore, 20 μL of the sample was deposited onto a copper grid that was coated with carbon, fixed for 15 min using 4% glutaraldehyde, and then negatively stained with 1 % uranyl acetate for 25 s. Following air drying at room temperature, transmission electron microscopy (TEM) was employed to examine the morphology of the fCA-BExo at an accelerating voltage of 90 kV. Detection of Protein Markers: A quantity of two milligrams of lyophilized fCA-BExo powder was lysed in 0.1 mL of RIPA lysis buffer, which contained 1% protease inhibitor and 1% phosphatase inhibitor, and the mixture was maintained on ice for 25 min. Following centrifugation at 12,500×g for 27 min, the supernatant was carefully collected, and the protein concentration was determined using the bicinchoninic acid assay. Equal protein amounts were then analyzed through SDS-PAGE electrophoresis, after which they were transferred onto NC membranes. These NC membranes were placed in a 4 °C environment overnight, gently shaken on a rocking platform while being exposed to primary antibodies that target, ALIX (dilution 1:2500), TSG101 (dilution 1:1500), CD9 (dilution 1:2500), and TOM70 (dilution 1:1500). After three washes with TBST at room temperature, secondary antibodies that were conjugated with HRP were introduced and gently agitated on a rocking platform for a duration of 2 h. The identification of the target protein bands was accomplished using ECL chemiluminescence imaging.

*Cell Proliferation and Cytotoxicity Assay*: BMSCs and RAW 264.7 cells were seeded in 96-well plates at a density of 10,000 cells/well and treated with f-CA(OH) solutions at varying concentrations (0.1–1.0 mg/mL) for 12 h. Subsequently, CCK-8 working solution (10% CCK-8 reagent in 90% DMEM medium) was added to each well. After incubating for 2 h at 37 °C, the absorbance was measured at 450 nm using a microplate reader to evaluate the effects of f-CA(OH) on cell proliferation and cytotoxicity.

*Cell Viability Assay*: BMSCs and RAW 264.7 cells were seeded in 6-well plates at a density of 1×10^6^ cells/well and treated with f-CA(OH) solutions at different concentrations (0, 0.1, and 1.0 mg/mL) for 12 h. After treatment, cells were stained with Calcein-AM/PI dual staining solution for 30 min at 37 °C. Fluorescence imaging was performed using an inverted fluorescence microscope to assess live (green, Calcein-AM) and dead (red, PI) cells. The ratio of viable cells was quantified to evaluate the cytotoxic

*Cellular Uptake Assay*: f-CA(OH) was labeled with FITC by stirring under light-protected conditions for 24 hours. Cells were seeded in confocal-specific culture dishes and incubated with f-CA(OH)-FITC working solution for 10 min, 30 min, 1 h, and 2 h, respectively. At each time point, the medium was discarded, and cells were washed three times with pre-cooled PBS. Nuclei were counterstained with DAPI staining solution under light-protected incubation for 15 min. Fluorescence imaging was performed using a super-resolution laser scanning confocal microscope (CLSM, Leica STELLARIS 5, Germany). Parallel samples were washed with PBS, digested with 0.25% trypsin, and filtered through a 300-mesh sieve. FITC fluorescence intensity was quantified via flow cytometry, with 10,000 cellular events acquired per sample.

*Intracellular ROS detection*: Intracellular ROS levels were quantified using DCFH-DA. The intense green fluorescence produced when DCFH-DA is oxidized by ROS in dicates intracellular ROS levels. RAW 264.7 cells were treated with different concentrations for 12 h and then incubated in serum-free medium containing DCFH-DA for 30 min. Imaging was performed using an inverted fluorescence microscope (Olympus IX73, Japan).

*JC-1 Quantitative Analysis*: RAW264.7 cells were seeded in 6-well plates at a density of 5×10⁵ cells per well. After the cells adhered, LPS (100 ng/mL) was added to simulate an inflammatory environment, and the cells were cultured under various treatment conditions for 24 hours. Subsequently, the cells were stained with JC-1 dye for 30 minutes in the dark. Changes in the mitochondrial membrane potential of RAW264.7 cells were then analyzed using confocal microscopy.

*Western Blot*: BMSC or RAW264.7 cells were seeded in 6-well plates at a density of 5×10⁵ cells per well and treated under different conditions for 24 h. After treatment, total cellular proteins were extracted using RIPA lysis buffer. The protein samples were separated by SDS-PAGE and then transferred onto PVDF membranes. The membranes were first blocked by shaking in a quick blocking solution for 1 h at room temperature. After blocking, the membranes were incubated overnight at 4 °C with primary antibodies (diluted according to the manufacturer’s instructions) under gentle shaking. The membranes were then washed three times for 10 minutes each and incubated with HRP-conjugated secondary antibodies (goat anti-rabbit IgG or anti-mouse IgG, diluted 1:1000 in washing buffer) for 1 hour at room temperature. Following three additional washes for 15 min each, the membranes were incubated with ECL detection reagent for 5 min, and then exposed using the Gel-View-6000Plus system (China). Finally, quantitative analysis of the gray values (GV) was performed using ImageJ software.

*Cell immunofluorescence staining*: After seeding the cells onto a confocal dish and applying the respective treatments, the cells were fixed in 4% paraformaldehyde for 15 min, then permeabilized with 0.5% Triton X-100 for 10 min. Blocking was performed with 1% BSA at room temperature for 1 h. The cells were then incubated overnight at 4 ℃ with the corresponding primary antibody. The following day, the cells were washed three times with phosphate-buffered saline (PBS), and secondary antibodies were added for 2 h at room temperature in the dark. Nuclei were counter stained with DAP. Fluorescence was observed using a confocal laser scanning microscope (CLSM, Leica STELLARIS 5, Germany), and representative.

*Osteoclast Induction Culture*: RAW 264.7 cells were grown in 6-well plates or in cell culture dishes with a diameter of 35 mm, at a density of 3×10^6^ cells for each well. When RAW 264.7 cells attained around 70% confluency, the existing medium was substituted with an osteoclast induction medium composed of high-glucose DMEM, 9% FBS, 5 nM RANKL/TNFSF11 protein, and 1.8 nM M-CSF protein, as well as the specified treatment conditions. The cells were maintained in culture for a total duration of 1 week and the induction medium was refreshed every 36 h.

*Osteogenic Induction Culture*: BMSC cells were cultivated in 6-well plates or 35 mm high-adhesion cell culture dishes at a density of 3×10^6^ cells per well. Once the cells attained around 65% confluency, the culture medium was replaced with an osteogenic induction medium consisting high-glucose DMEM, 9% FBS, 110 nM dexamethasone, 12 mM β-glycerophosphate disodium salt hydrate, and 55 μM L-ascorbic acid, along with the appropriate treatment conditions. The induction medium was refreshed every two days, and the cells were maintained in culture for either 7 or 14 days.

*ALP Staining*: On the seventh day of osteogenic induction, the osteogenic induction medium was discarded, and the cells underwent three washes with prechilled PBS. Following this, the cells were fixed at room temperature using a 4% paraformaldehyde solution for 15 min. The ALP staining working solution, made according to the kit guidelines, was applied, and the cells were incubated at 37°C for 30 min. To halt the reaction, the cells were rinsed three times with distilled water. The formation of blue-purple precipitates was then observed using an inverted microscope (Olympus IX73, Japan).

*Alizarin Red S (ARS) Mineralized Nodule Staining*: On the fourteenth day of osteogenic induction, the osteogenic differentiation medium was removed, and the cells were washed with prechilled PBS five times. Subsequently, approximately 500 μL of the Alizarin Red S staining solution (2%, pH 4.2) was used to cover the cellular layer, and staining was carried out at room temperature for a duration of 25 min. To eliminate non-specific staining, the cells were washed multiple times with distilled water until no red dye could be detected in the wash solution. Following air-drying at room temperature, an inverted microscope (Olympus IX73, Japan) was used to evaluate the formation of red mineralized nodules.

*Micro-CT Imaging and Quantification*: Before euthanizing the animals, their femurs underwent scanning with a Metis™ PET-CT system. The parameters for the scan included a maximum of 740 spiral projections, a voltage setting of 70 kV, and an exposure duration of 300 ms. Subsequently, the collected data were loaded into 3D Slicer- software for the purpose of three-dimensional reconstruction, allowing the calculation of relevant indices related to the bone tissue.

*Evaluation of Targeting Specificity of f-CA-BExo*: f-CA-BExo (80 μg/mL, 150 μL) was labeled with a Cy5 dye solution (1 mM/mL) and intravenously injected via the mouse tail vein. An in vivo small animal imaging system was used to observe the targeting of f-CA-BExo to the femoral bone marrow cavity of OVX mice and its metabolic pathway at 2, 4, 8, 12, and 24 hours post-injection.

*Enzyme-linked Immunosorbent Assay (ELISA)*: The secretion levels of cytokines TNF-α, IL-1β, IL-6, IL-10, TGF-β1, CTX-1 and PINP in cell culture supernatants and animal samples were measured using an ELISA kit, following the manufacturer’s instructions.

*Statistical Analysis*: The data are presented as mean ± standard deviation (SD). To assess statistical significance among the sample groups, one-way ANOVA was conducted using GraphPad Prism 10 software. The p-values are reported as follows: ns≥0.05, *p<0.05, **p<0.01, ***p<0.001, ****p<0.0001. Flow cytometry data were processed with FlowJo version 10.8.1, and ImageJ version 1.4.3.67 was used for the analysis of fluorescent grayscale images.


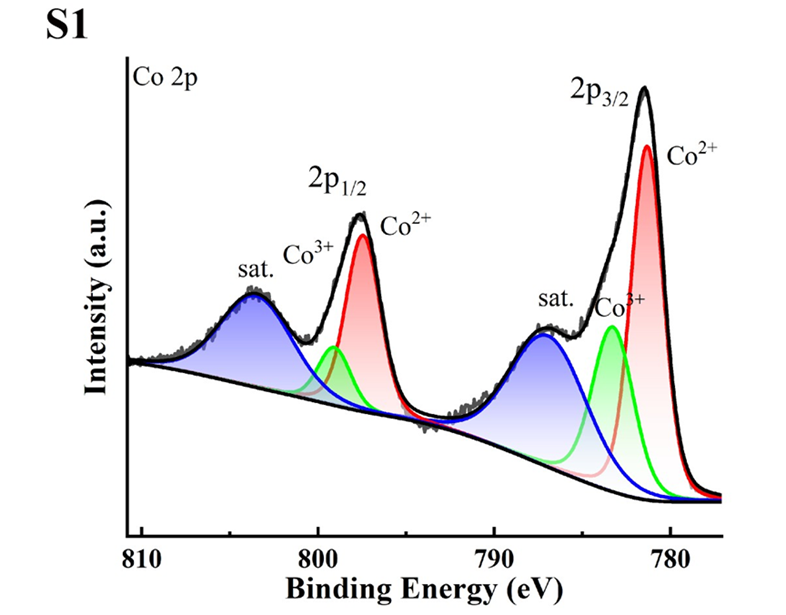


**Figure S1.** High-resolution XPS spectrum of Co 2p.


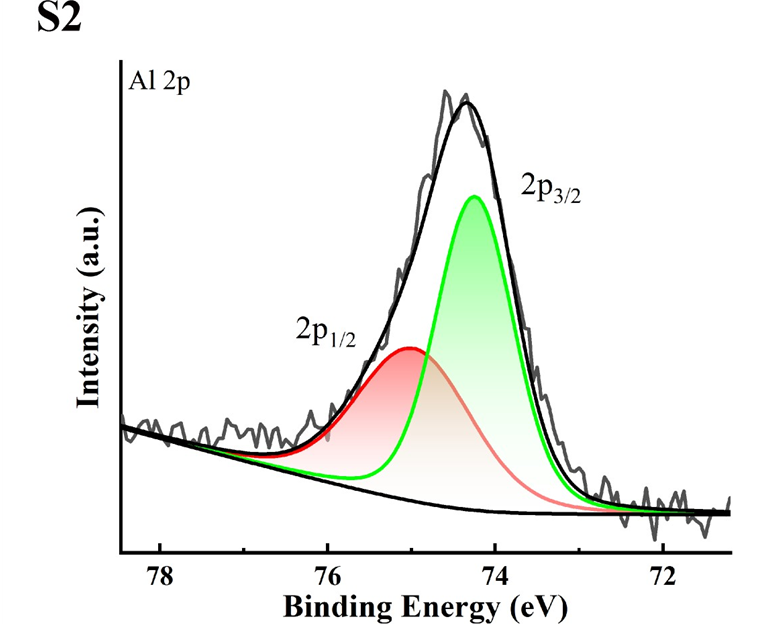


**Figure S2.** High-resolution XPS spectrum of Al 2p.


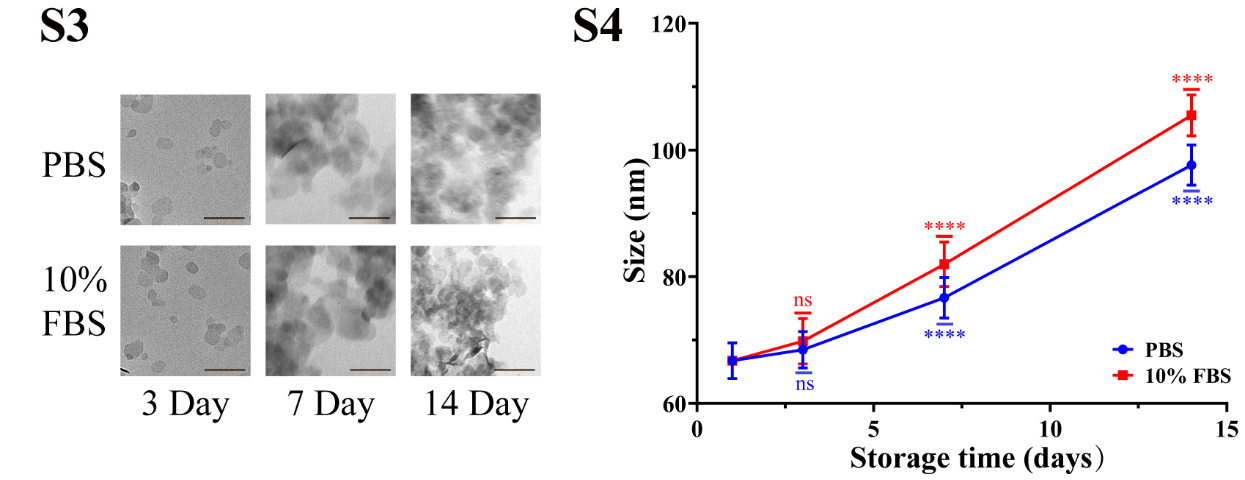


**Figure S3.** TEM images of nanosheets under different storage conditions and durations. (Scale bar: 100 nm).

**Figure S4.** Diameter variation of nanosheets under different storage conditions over time.


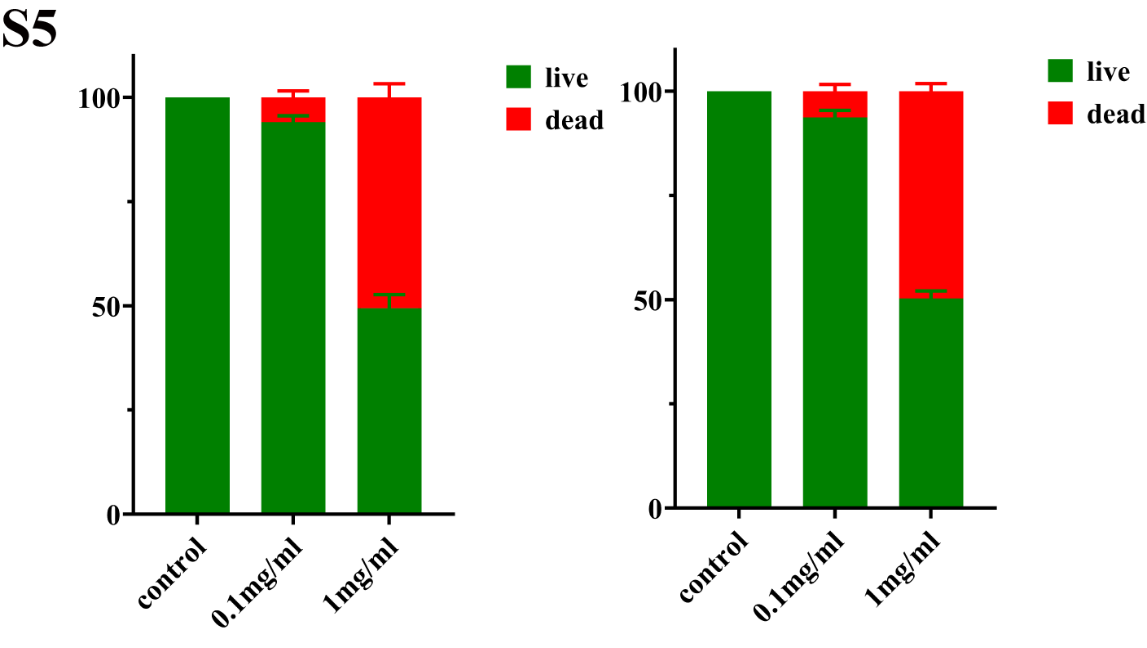


**Figure S5.** Live/dead staining of BMSCs and RAW264.7 cells following co-culture with varying concentrations of fCA(OH). Data are presented as mean ± standard error.


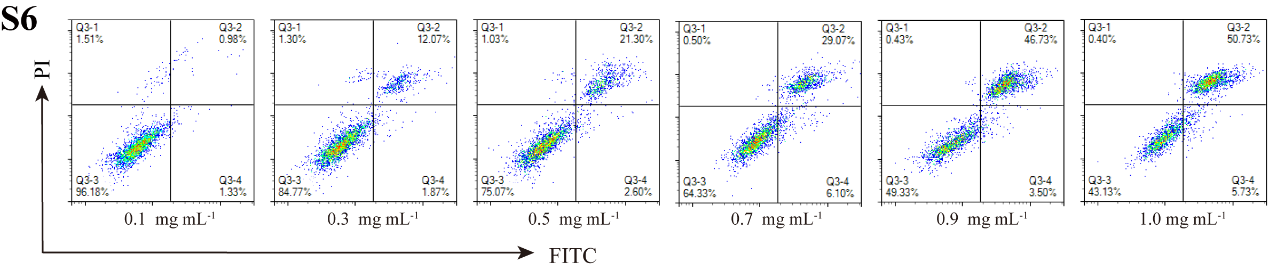


**Figure S6.** Flow cytometry analysis to evaluate apoptosis in BMSC cells treated with different concentrations of f-CA(OH) (0.1, 0.3, 0.5, 0.7, 0.9, and 1.0 mg/mL f-CA(OH)).


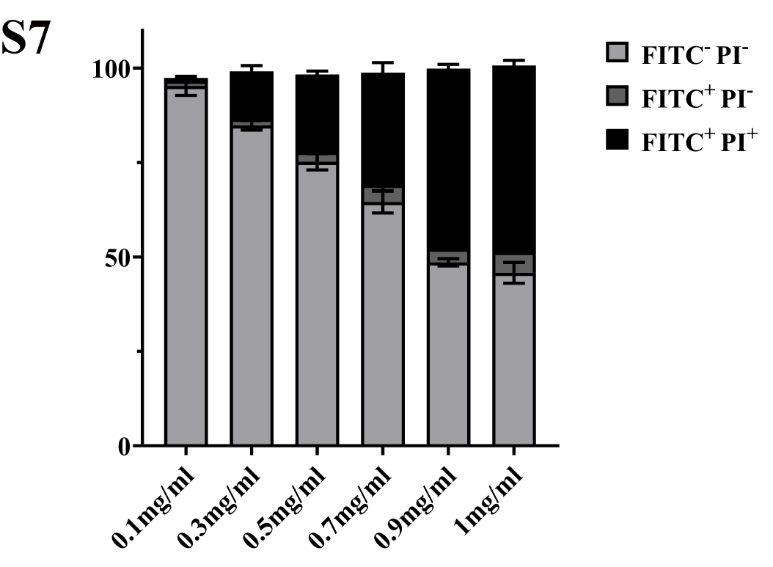


**Figure S7.** Apoptosis analysis of BMSCs following co-culture with varying concentrations of fCA(OH). Data are presented as mean ± standard error.


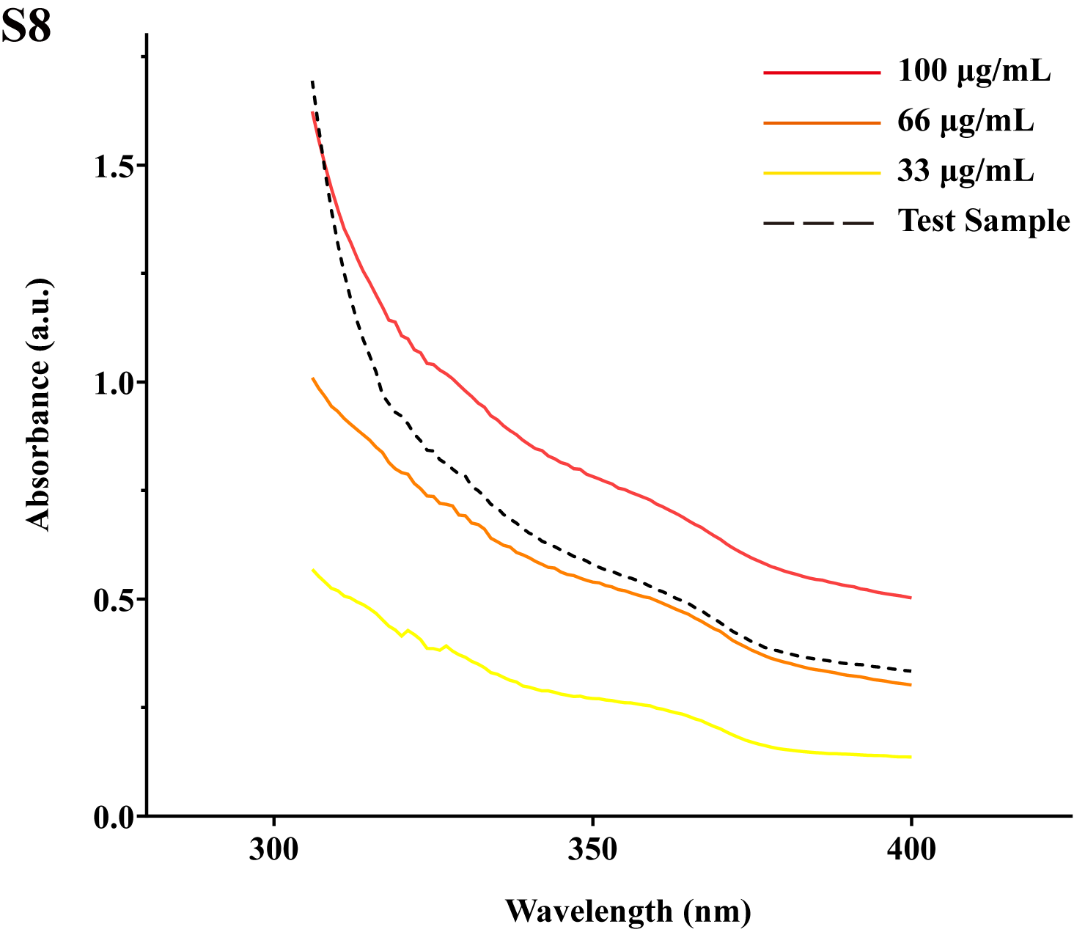


**Figure S8.** Quantification of residual f-CA(OH) in exosomal suspension by UV-Vis spectroscopy (68 μg/mL).


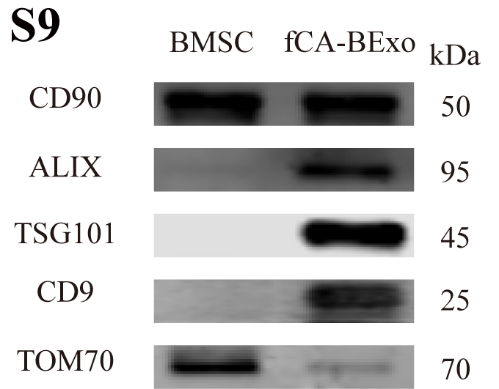


**Figure S9.** Western blot analysis of exosomal markers and mitochondrial contamination. Exosomes were isolated from the co-culture system and lysed for protein extraction. Equal amounts of exosomal and whole-cell protein were probed with antibodies against exosomal markers (CD9, CD90, ALIX, TSG101) and mitochondrial contamination marker TOM70.


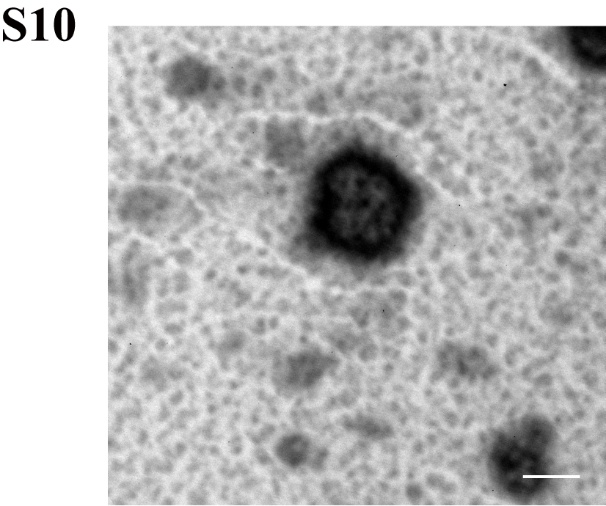


**Figure S10.** Transmission electron microscopy (TEM) analysis of engineered exosomes. Engineered exosomes were purified from the co-culture system by ultracentrifugation. (Scale bar: 50 nm)


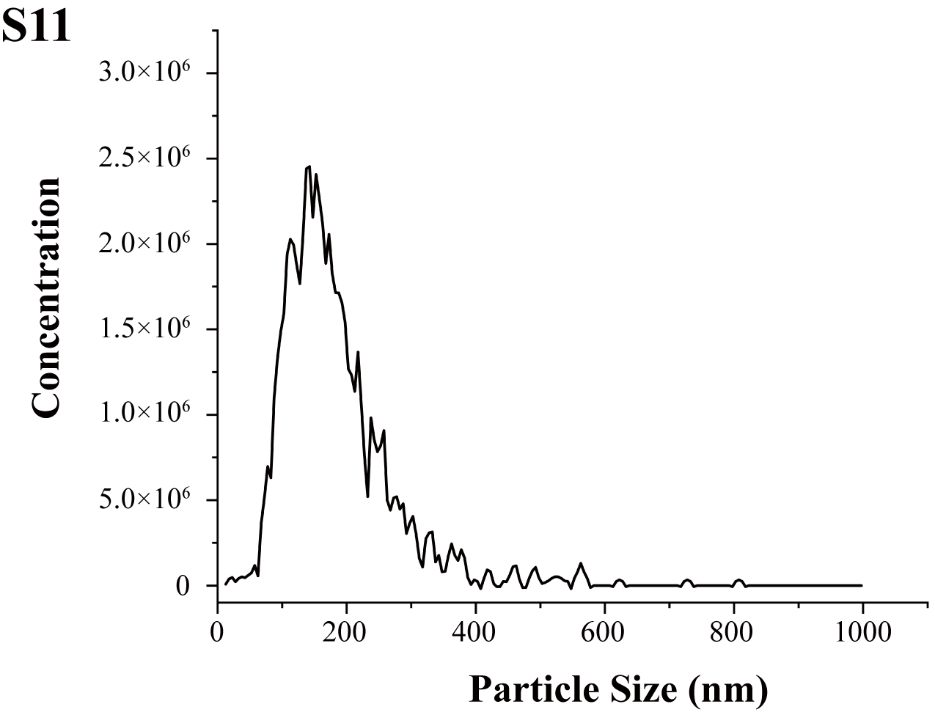


**Figure S11.** Nanoparticle tracking analysis (NTA) of engineered exosomes. Engineered exosomes were isolated from the co-culture system by ultracentrifugation. Hydrodynamic diameter was measured using a NTA with triplicate samples.


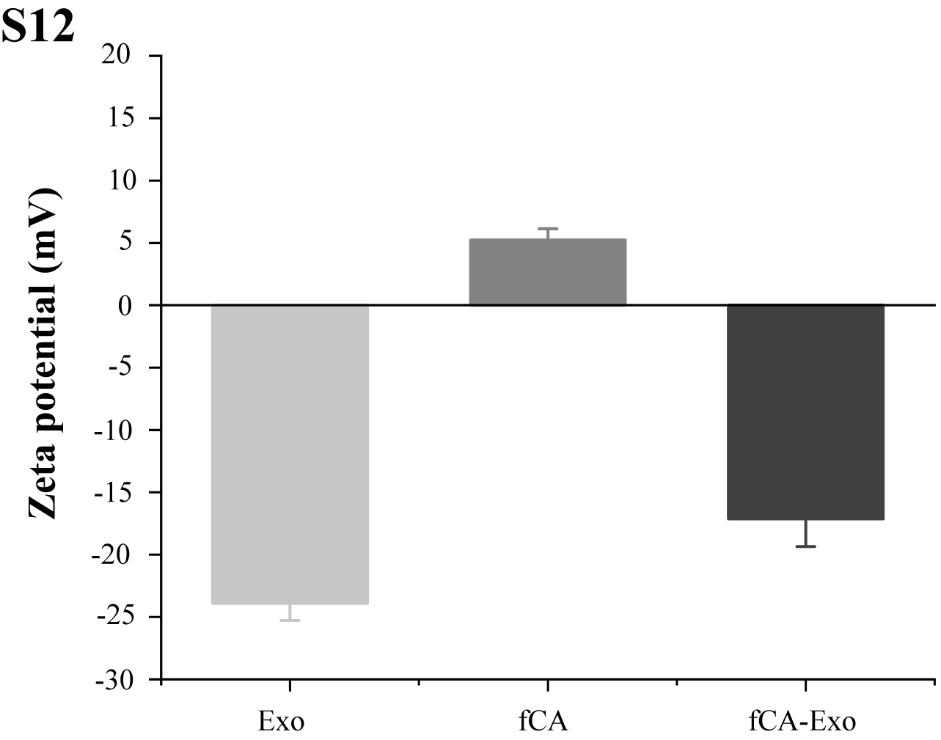


**Figure S12.** Zeta potential analysis of engineered exosomes. Engineered exosomes exhibited a zeta potential of –17.1 ± 1.3 mV, significantly less negative than conventional exosomes. Data represent mean ± SD (n = 3 independent measurements).


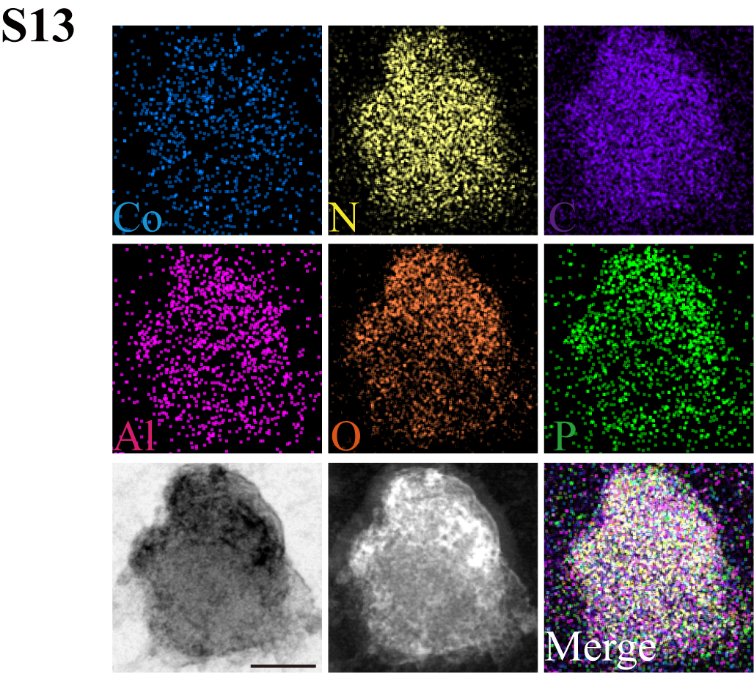


**Figure S13.** Elemental mapping of Co, Al, C, O, N and P in fCA-Exo. (Scale bar: 50 nm).


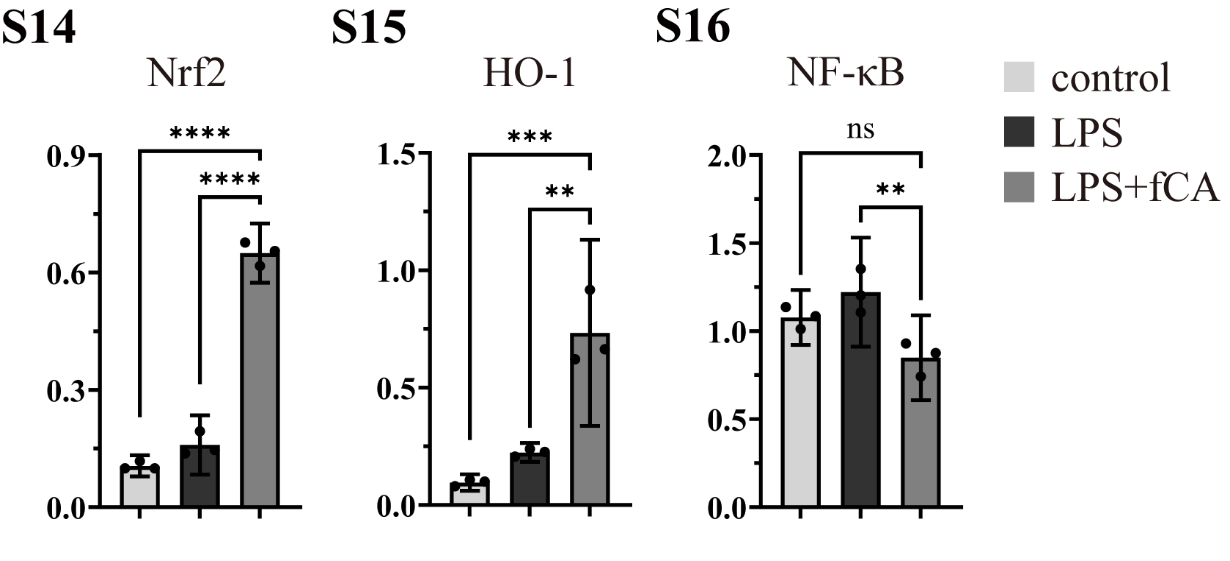


**Figure S14.** Western blot analysis of Nrf2 expression. Nuclear Nrf2 expression in LPS-stimulated macrophages treated with f-CA(OH).

**Figure S15.** Western blot analysis of HO-1 expression. Upregulation of HO-1 protein expression by f-CA(OH). HO-1 levels in f-CA(OH)-treated macrophages under LPS-induced oxidative stress.

**Figure S16.** Western blot analysis of NF-κB expression. Suppression of NF-κB nuclear translocation by f-CA(OH).

Statistical significance was determined by one-way ANOVA with Tukey’s post-hoc test. Data are presented as mean ± standard error. **p < 0.01, ***p < 0.001, ****p < 0.0001.


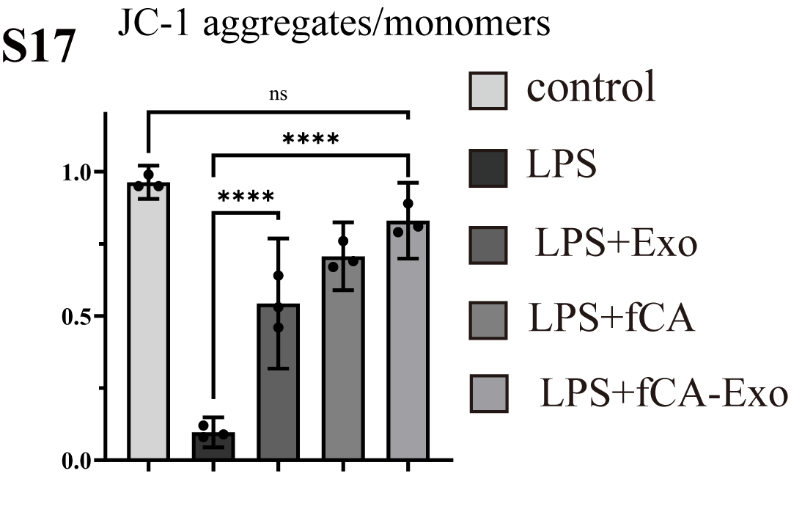


**Figure S17.** JC-1 fluorescence intensity ratio (aggregates/monomers) analysis of mitochondrial membrane potential. Statistical significance was determined by one-way ANOVA with Tukey’s post-hoc test. Data are presented as mean ± standard error. *p < 0.05, **p < 0.01, ***p < 0.001, ****p < 0.0001.


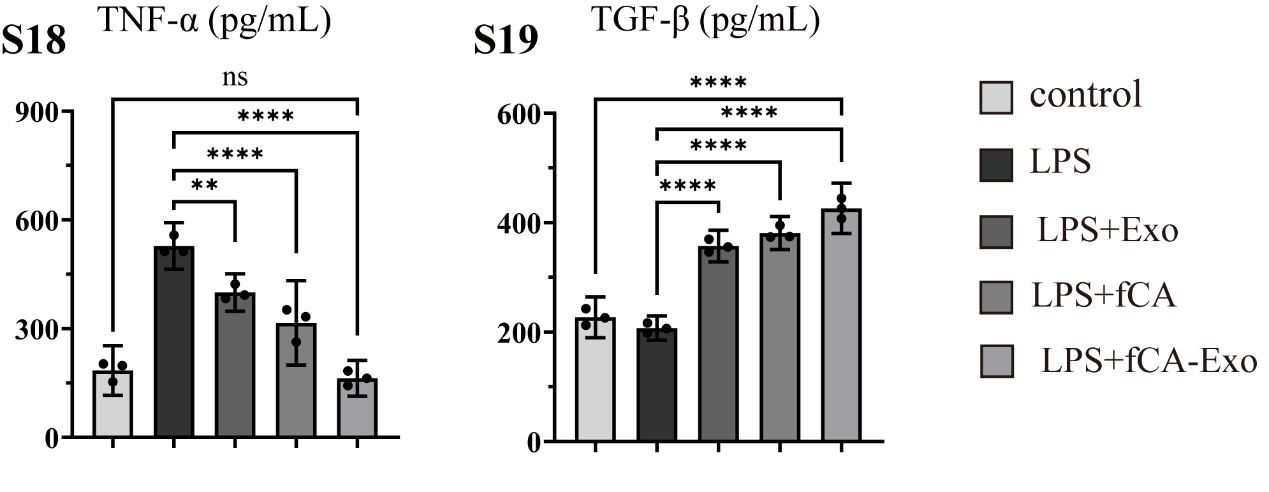


**Figure S18.** TNF-α levels in cell supernatant (ELISA). Quantified using human/mouse TGF-β ELISA kit.

**Figure S19.** TGF-β levels in cell supernatant (ELISA). Quantified using human/mouse TGF-β ELISA kit.

Statistical significance was determined by one-way ANOVA with Tukey’s post-hoc test. Data are presented as mean ± standard error. *p < 0.05, **p < 0.01, ***p < 0.001, ****p < 0.0001.


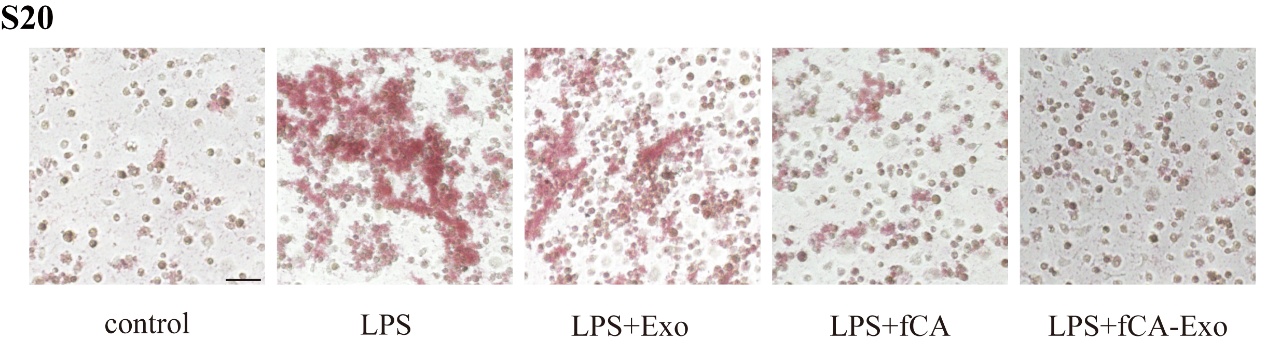


**Figure S20.** TRAP staining of osteoclast differentiation from monocytes. (scale bar: 100 μm.)


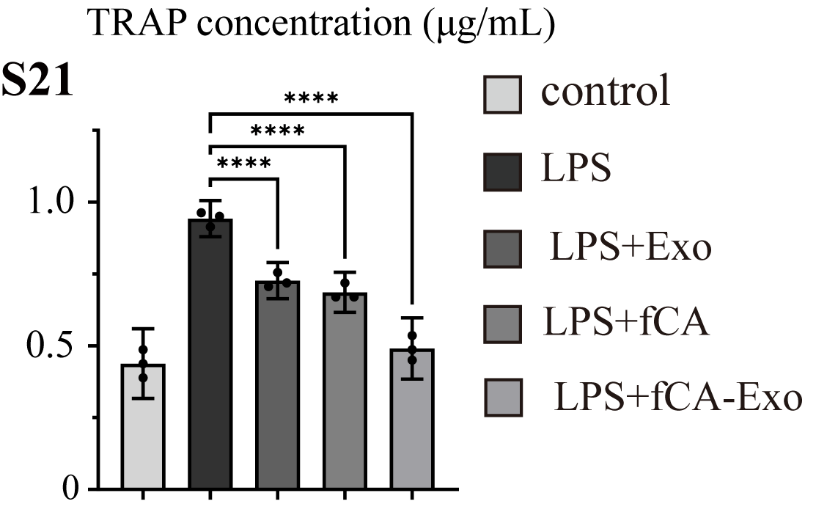


**Figure S21.** TRAP activity in cell supernatant (colorimetric assay).Quantified using a commercial TRAP activity assay kit with pNPP substrate (absorbance measured at 405 nm). Statistical significance was determined by one-way ANOVA with Tukey’s post-hoc test. Data are presented as mean ± standard error. *p < 0.05, **p < 0.01, ***p < 0.001, ****p < 0.0001.


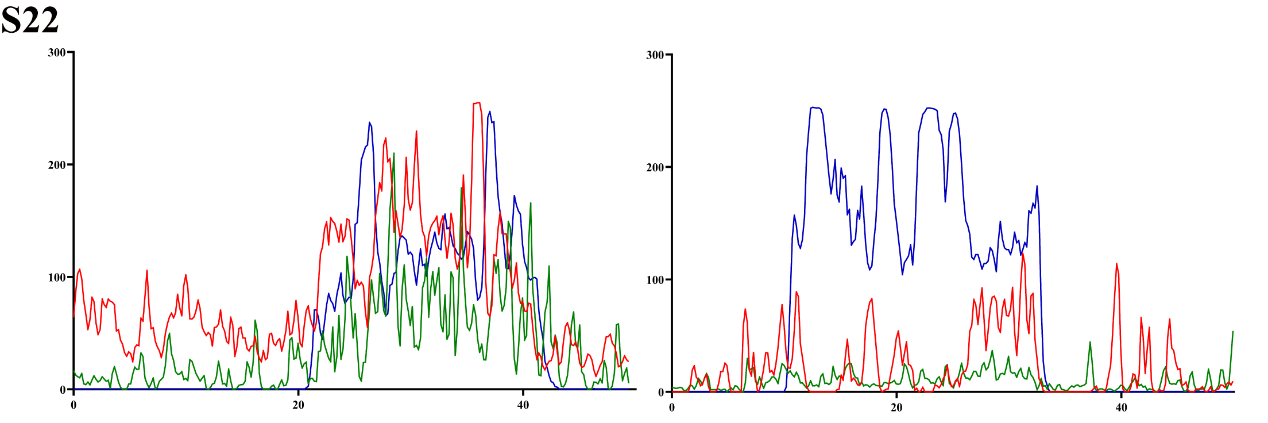


**Figure S22.** Immunofluorescence co-localization and fluorescence intensity analysis of PI3K and p-AKT. Cells were stained with anti-PI3K (green) and anti-p-AKT (red) antibodies, counterstained with DAPI (blue, nuclei). Fluorescence co-localization (yellow regions in merged images) and intensity profiles were analyzed using ImageJ.


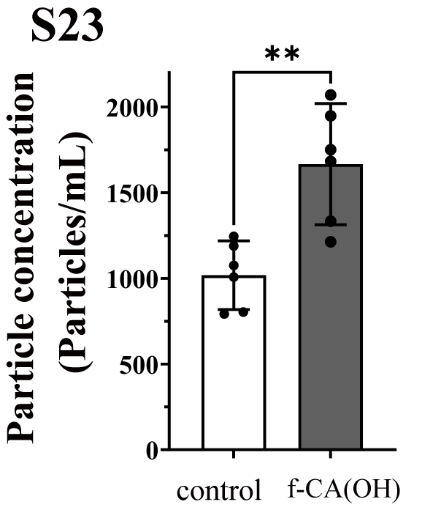


**Figure S23.** Particle concentration distribution measured by nanoparticle tracking analysis (NTA).Data are presented as mean ± standard error. *p < 0.05, **p < 0.01, ***p < 0.001, ****p < 0.0001.


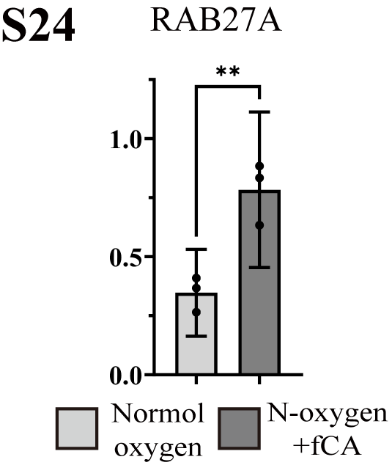


**Figure S24.** Western blot analysis of RAB27A expression. Statistical significance was determined by one-way ANOVA with Tukey’s post-hoc test. Data are presented as mean ± standard error. *p < 0.05, **p < 0.01, ***p < 0.001, ****p < 0.0001.


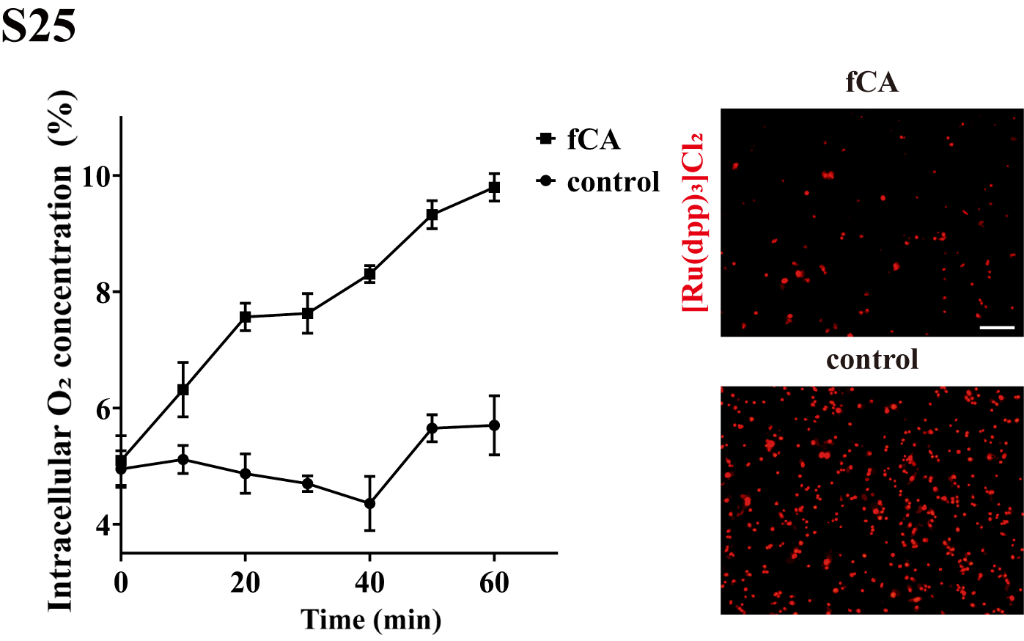


**Figure S25.** Material-induced increase in intracellular oxygen concentration detected with Ru(dpp)₃Cl₂. Left: intracellular oxygen concentration calculated from fluorescence measured by a microplate reader after Ru(dpp)₃Cl₂ staining. Right: representative fluorescence micrographs acquired 30 min after material addition (scale bar: 50 μm). Data are presented as mean ± standard error.


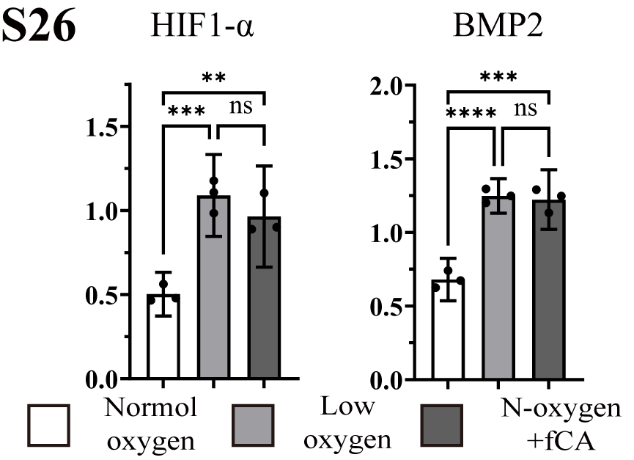


**Figure S26.** Western blot analysis of HIF1-α and BMP 2 expression. Statistical significance was determined by one-way ANOVA with Tukey’s post-hoc test. Data are presented as mean ± standard error. *p < 0.05, **p < 0.01, ***p < 0.001, ****p < 0.0001.


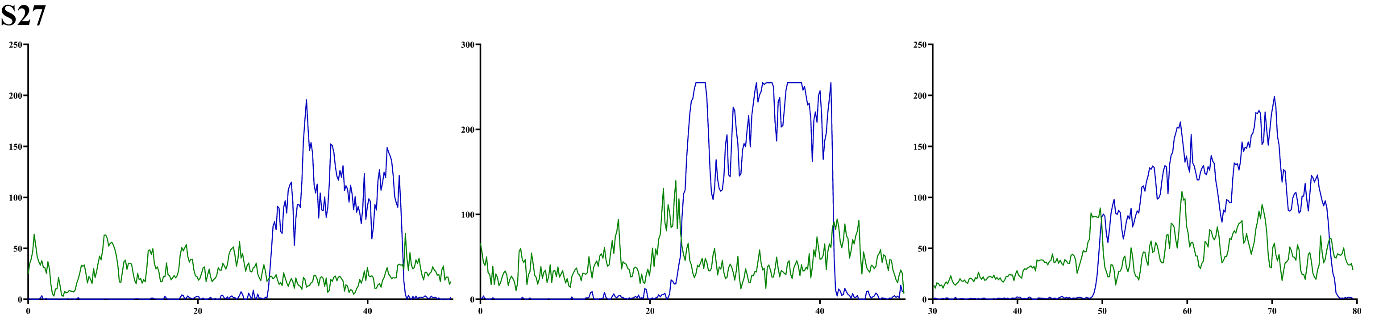


**Figure S27.** Immunofluorescence co-localization and fluorescence intensity analysis of HIF1-α. Cells were stained with anti- HIF1-α (green) counterstained with DAPI (blue, nuclei). Fluorescence co-localization and intensity profiles were analyzed using ImageJ.


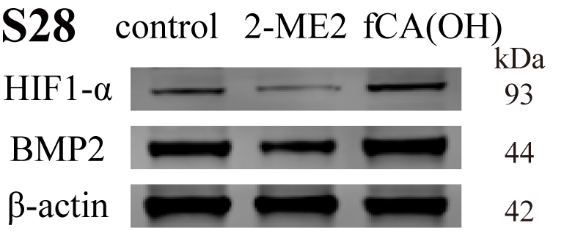


**Figure S28.** Western blot analysis of 2-ME2 blocks f-CA(OH)-dependent BMP2 expression via HIF1-α inhibition.


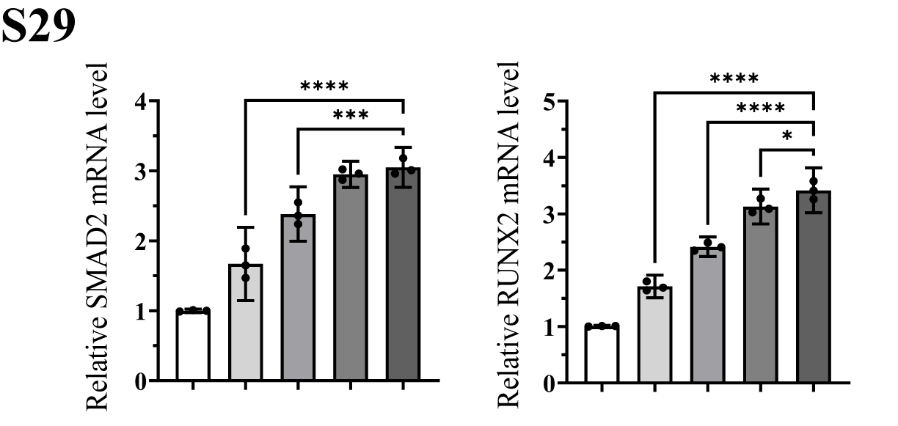


**Figure S29.** qRT-PCR analysis of SMAD2 and RUNX2 mRNA expression in BMSC cells. Data are presented as mean ± standard error. *p < 0.05, **p < 0.01, ***p < 0.001, ****p < 0.0001.


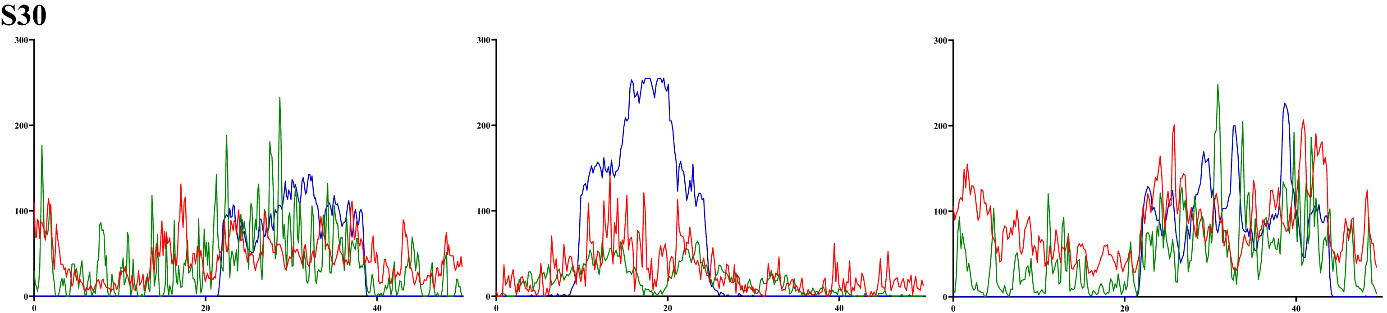


**Figure S30.** Immunofluorescence co-localization and fluorescence intensity analysis of SAMD 2 and RUNX 2. Cells were stained with anti- RUNX 2 (green) and anti- SAMD 2 (red) antibodies, counterstained with DAPI (blue, nuclei). Fluorescence co-localization (yellow regions in merged images) and intensity profiles were analyzed using ImageJ.


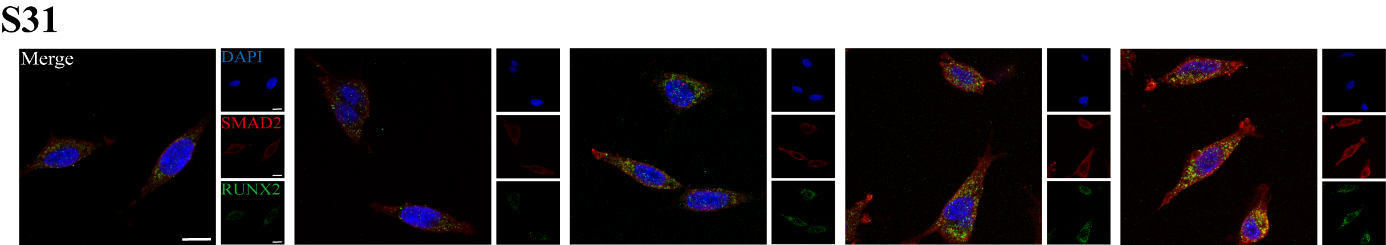


**Figure S31.** CLSM images displaying the subcellular localization and immunofluorescence expression of SMAD2 and RUNX2 in MC3T3-E1 cells (scale bar: 10 μm).


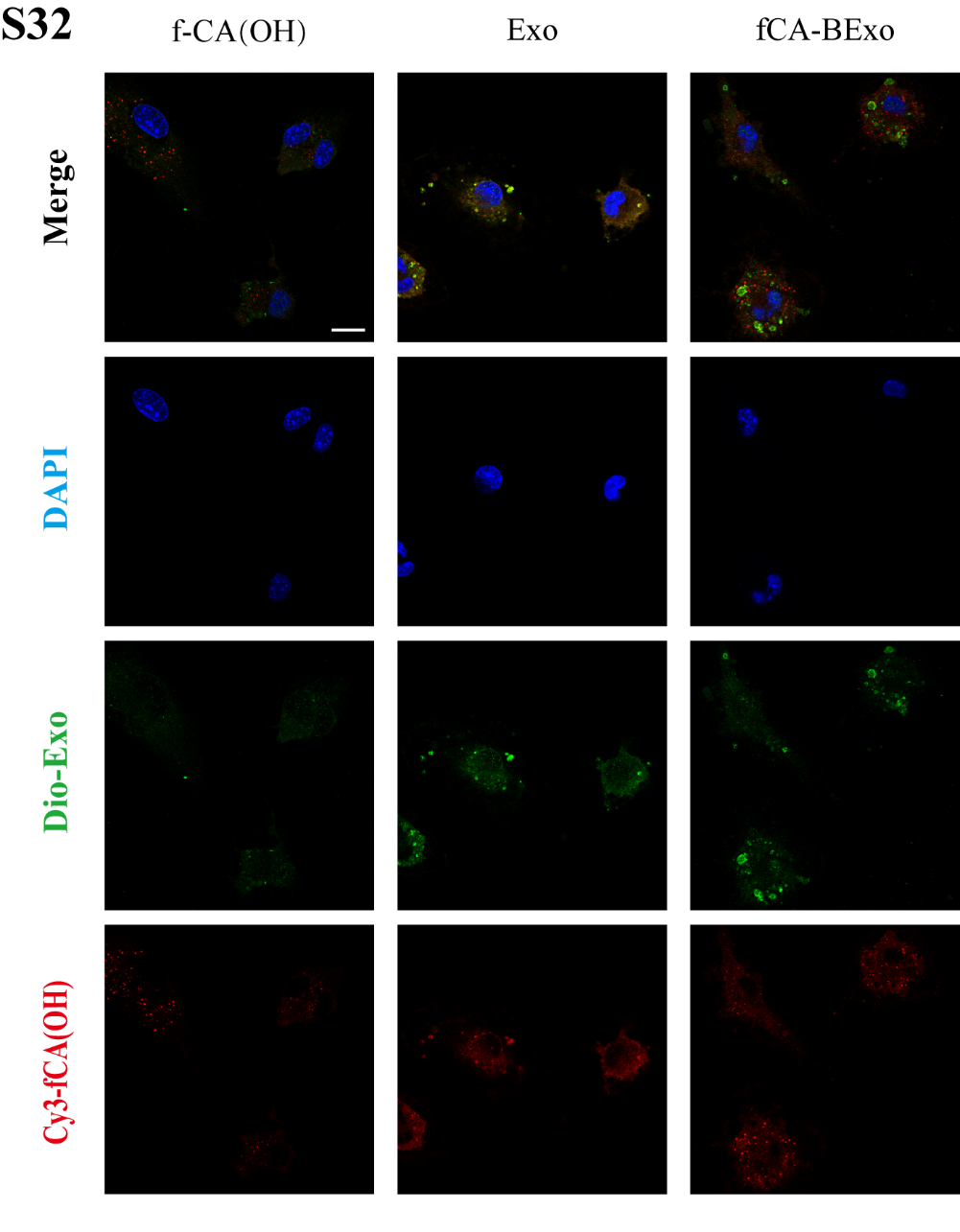


**Figure S32.** Cellular uptake of DiO-labeled exosomes and release of f-CA(OH) nanosheets in isolated marrow cells (scale bar: 10 μm).


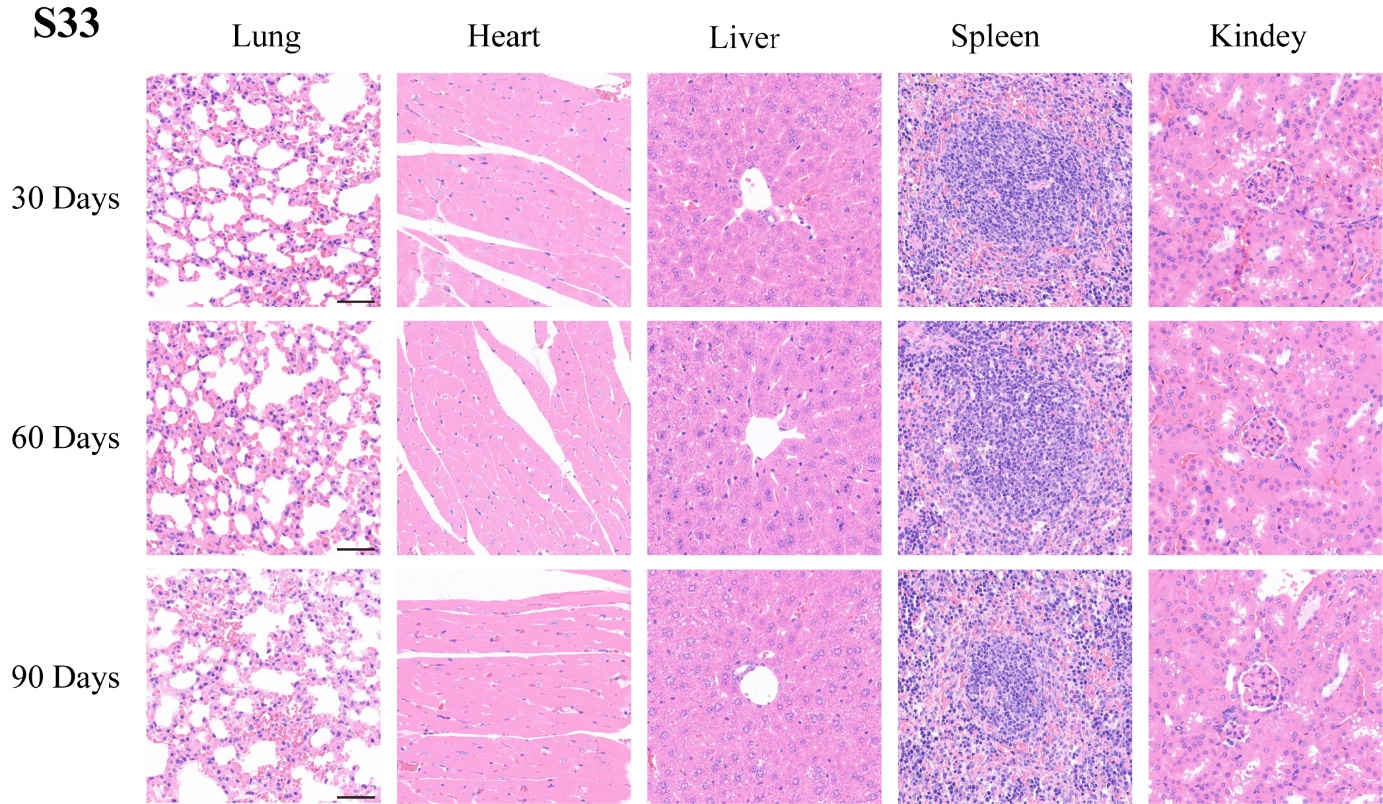


**Figure S33.** Histopathological evaluation of major organs (heart, liver, spleen, lung, kidney) after 30, 60, and 90 days of treatment with fCA-BExo (scale bar: 50 μm).


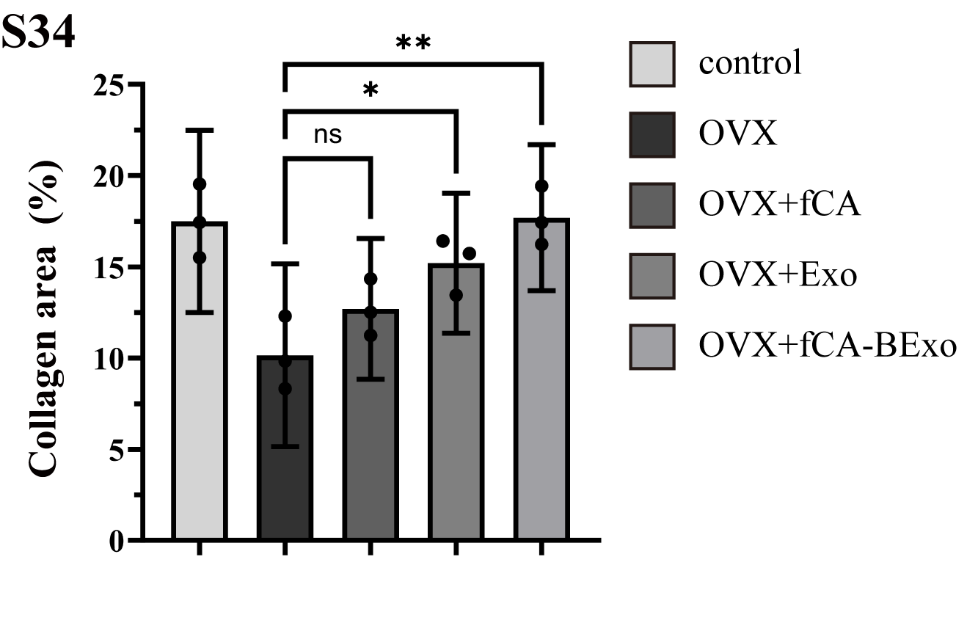


**Figure S34.** Quantitative analysis of collagen fiber deposition in femur by Masson's trichrome staining. Data are presented as mean ± standard error. *p < 0.05, **p < 0.01, ***p < 0.001, ****p < 0.0001.


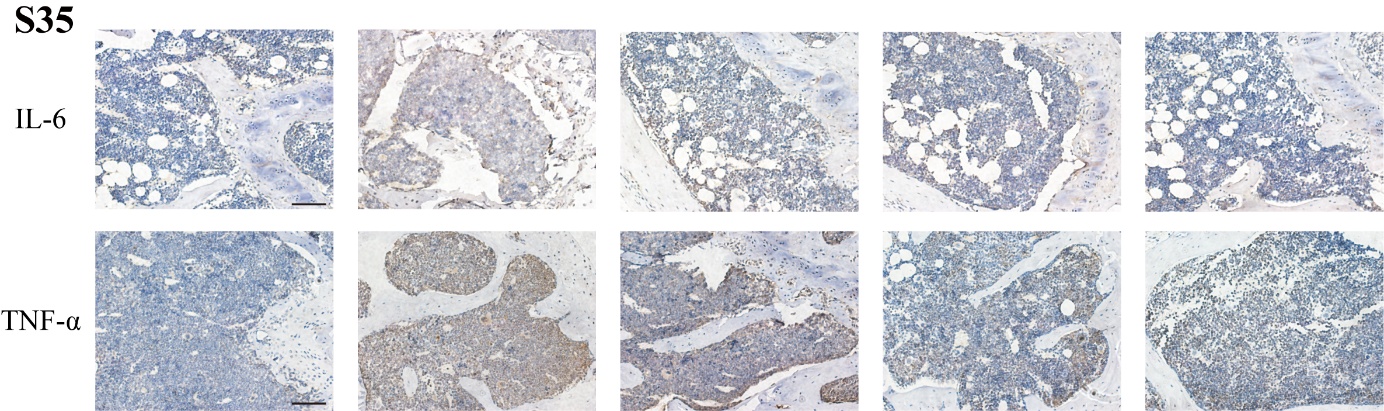


**Figure S35.** Immunohistochemical (IHC) staining of IL-6 and TNF-α expression in bone marrow (scale bar: 50 μm). (scale bar: 50 μm).


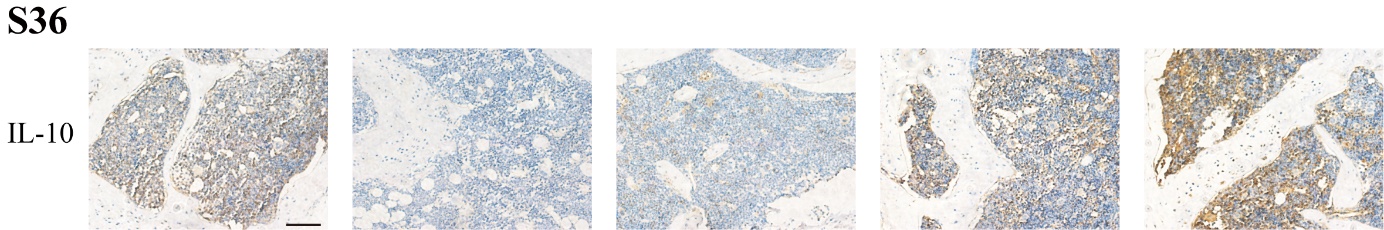


**Figure S36.** Immunohistochemical (IHC) staining of IL-10 expression in bone marrow (scale bar: 50 μm). (scale bar: 50 μm).


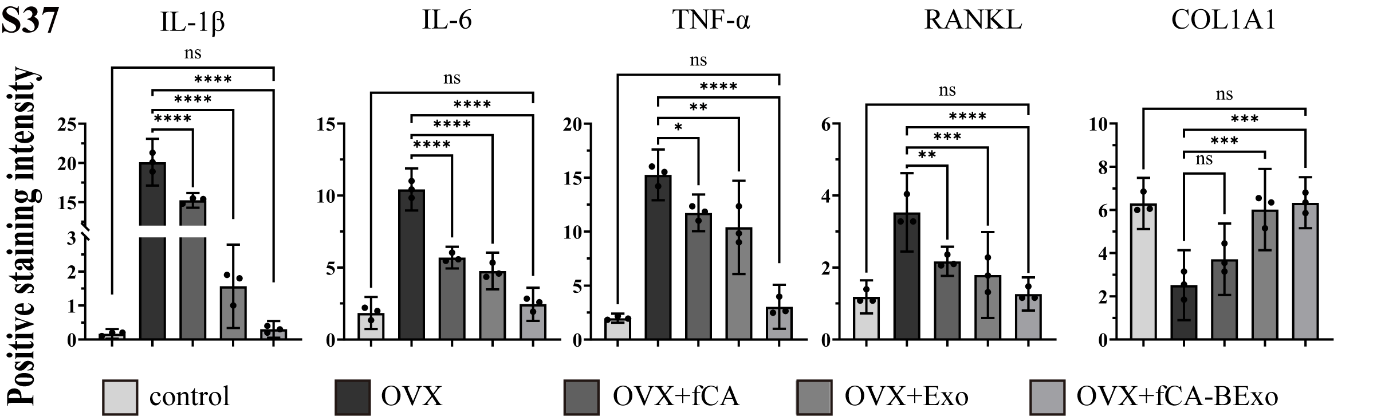


**Figure S37.** Semi-quantitative analysis of immunohistochemical staining for inflammatory factors, osteoclastogenesis and osteogenesis markers in femoral tissues. Data are presented as mean ± standard error. *p < 0.05, **p < 0.01, ***p < 0.001, ****p < 0.0001.


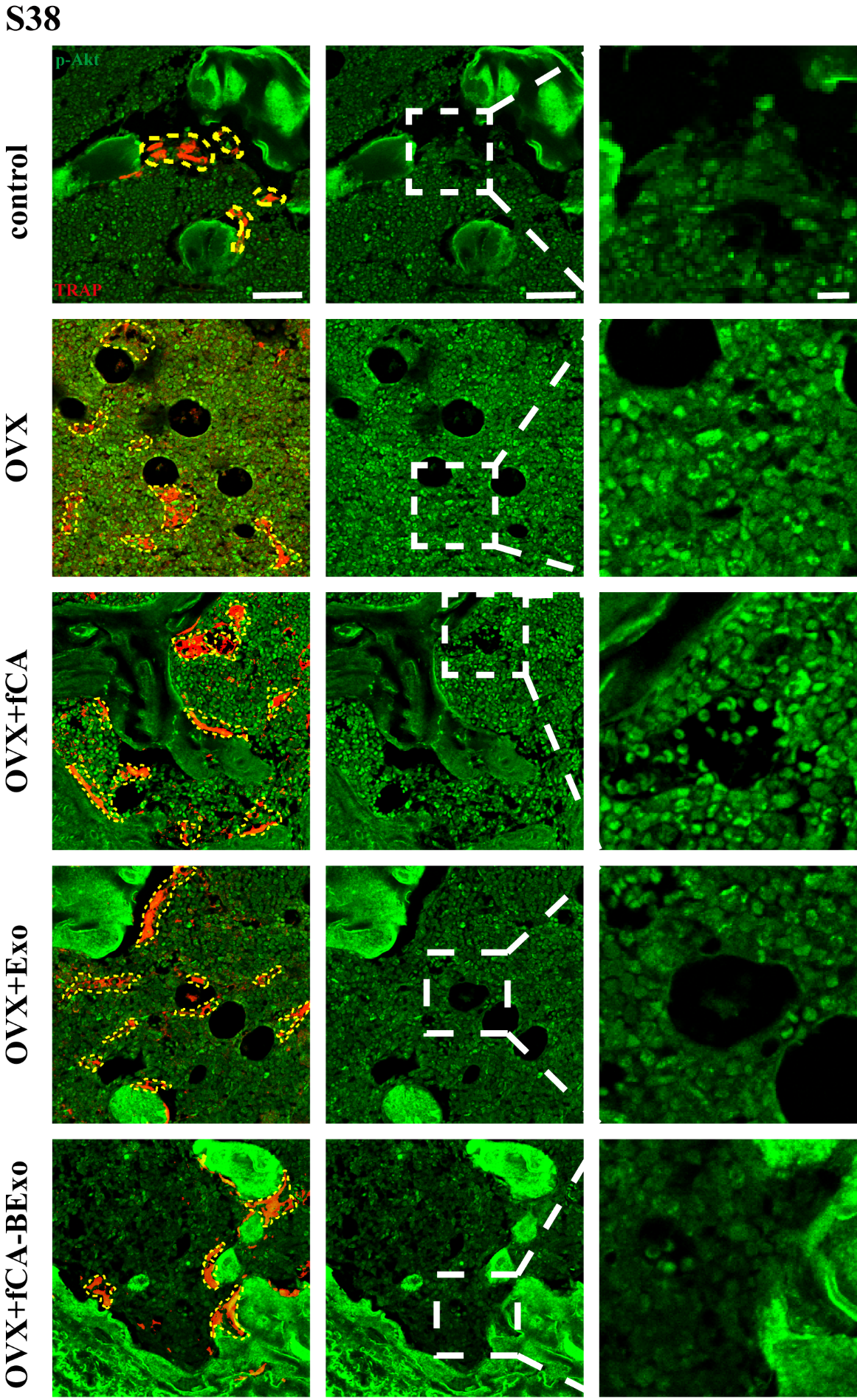


**Figure S38.** Inhibition of PI3K-AKT signaling in osteoclasts by fCA-BExo in OVX mice (red: TRAP; green: p-Akt; scale bars: 50 μm (low magnification), 10 μm (high magnification)).
